# High-Density, Extrudable Collagen Types I and II Microfiber-Based Inks for Tissue Engineering Applications

**DOI:** 10.3390/polym18182261

**Published:** 2026-09-16

**Authors:** Kevin Capariño, Shu-Tung Li

**Affiliations:** Shu-Tung and Alice Li Foundation, Laboratory for Tissue Engineering Research, Oakland, NJ 07436, USA

**Keywords:** biomaterials, tissue engineering, 3D bioprinting, collagen-based ink, type I collagen, type II collagen, extracellular matrix, collagen scaffolds, mechanical properties, cell compatibility

## Abstract

Extrusion-based 3D-printing of collagen currently relies almost exclusively on solubilizing collagen, restricting ink formulations to low-density hydrogels that typically contain less than 1% (10 mg/mL) collagen. This low collagen content results in poor mechanical properties, which limit print stability at room temperature and pose significant challenges for engineering scaffolds for high load-bearing tissues. Here, we introduce a method that leverages collagen’s electrostatic properties to generate extrudable inks composed of intact, insoluble collagen across a broad range of densities, including 20% (200 mg/mL). These inks preserve native collagen fiber architecture, can be prepared at neutral pH, and allow for room-temperature printing without additional stabilization procedures. Our approach is applicable to both type I collagen and type II collagen, enabling scaffolds to be fabricated with collagen in a form and at contents that more closely reflect those of a variety of tissues. By overcoming limitations associated with soluble, atelocollagen hydrogels, these high-density, insoluble collagen microfiber-based inks provide a new platform for developing physiologically relevant scaffolds for tissue-engineering applications.

## 1. Introduction

Collagen is the most abundant protein in all mammals. By using well-established methods of collagen isolation and purification [1,2,3,4], large quantities of fiber-forming collagens (particularly type I, type II, and type III collagen) can be obtained from tissues harvested from the abattoirs such as bone (type I), skin (type I and type III), tendon (type I), cartilage (type II), and ligament (type I). In particular, intact, insoluble type I collagen from animal tendon and corium of the hide have been one of the most popular natural biopolymer materials used in tissue engineering and regenerative medicine applications [1,5]. Conventional type I collagen-based implants and medical products, prepared through reconstitution and engineering of intact, insoluble type I collagen, have been proven to be safe and effective for guiding the regeneration of injured and diseased tissues for over three decades [1], and remain widely used today with common formats including high-density membranes, porous sponges, and tubular matrices [1].

Though type II collagen isolation and purification have already been described from articular cartilage, sterna and trachea [3,4,6], there has been an absence of implants prepared from intact, insoluble type II collagen in the clinic. Pieper et al. reported that native type II collagen does not properly swell in acid [6], thus making collagen reconstitution and engineering techniques possible for intact, insoluble type I collagen to not be applicable for intact, insoluble type II collagen. Instead, the purified type II collagen material is solubilized, allowing the resulting type II collagen monomers to be dissolved in acidic solutions for subsequent use in the production of sponges, fibrillated gels, or directly incorporated in material composites [7]. Even type I collagen is used as a substitute when a collagen-based scaffold is desired for applications intended for type II collagen, such as articular cartilage in the knee [8,9]. Thus, a unique need exists for intact, insoluble type II collagen-based scaffolds for applications involving type II collagen-rich tissues.

While type I collagen and type II collagen-based scaffolds briefly described above are being evaluated in combination with cells and growth factors for difficult to heal tissues [7,8,10,11,12], 3D bioprinting has simultaneously provided innovative options for fabricating 3D matrices. These matrices can either be a standalone extracellular matrix (ECM) scaffold printed from natural or synthetic biomaterials, or these matrices can be what we may consider a tissue-equivalent implant in composition when the “ink” includes cells and/or bioactive molecules [13]. Due to the programming capabilities of 3D-printer software, material distribution can be precisely controlled so that printed matrices can feature native tissue structures and architecture. This capability is not readily achievable when engineering macroscopic, reconstituted collagen fibers into scaffolds. Instead, 3D-printing approaches leverage the shear-thinning properties of hydrogels to facilitate collagen extrusion [14,15,16,17,18,19]. However, collagen’s unique physical–chemical properties have precluded the development of suitable collagen-based inks for certain bioprinting applications.

Typically when preparing hydrogels, collagen is first pepsin-digested to obtain soluble monomers. The natural solubility of collagen monomers or small collagen molecular aggregates in acidic solutions then limits ink formulations to rarely exceed 1% (10 mg/mL) [18,19,20], a density too low to recapitulate the native collagen contents of tissues especially in weight-bearing regions of the body [21,22,23,24]. Collagen hydrogel-based inks would then require extra stabilization steps during the extrusion-printing process to mitigate the risk for structural collapse due to weak mechanical properties [18,19,25,26,27,28,29,30]. Another aspect of collagen to be mindful of when extruding is that collagen precipitates (coacervates) near neutral pH (the isoelectric point of native collagen), at body temperature, or in the presence of salt (e.g., NaCl) to form fibers [26,27] that may prevent the flow of collagen through extrusion nozzles if these variables are not carefully controlled.

It is well recognized that increasing the collagen content of an ink is the most direct and effective way to enhance its mechanical properties and, consequently, improve the printability of collagen-based formulations [18,26,31,32,33,34,35]. Recent advancements in preparing inks from soluble collagen include achieving densities exceeding 2% (20 mg/mL), where commercial options are available up to 8% (80 mg/mL) [31,32,33]. Though less common, inks composed of collagen that did not undergo pepsin treatment have also demonstrated enhanced printability at higher collagen densities, with one example successfully attempting 12.5% (125 mg/mL) [34,35]. However, attempts still rely on temperature considerations or stabilization steps as part of their collagen printing workflow, and current achievable collagen densities can still be improved to better resemble those of weight-bearing tissues (e.g., ~20% collagen wet weight in meniscus [24]).

In contrast to previously reported highly concentrated collagen inks, we developed novel collagen inks composed of intact, insoluble collagen microfibers that can be prepared, maintained, and extruded at room temperature at various collagen densities (including 20% (200 mg/mL)) without explicit temperature control or additional stabilization procedures such as printing into a support material [26,30] or simultaneous photocrosslinking during extrusion [18,27,28]. These inks enable straightforward fabrication of collagen-based scaffolds in which collagen is preserved in its native form, and is present at concentrations comparable to the range of most collagen-rich tissues and organs. Since the preparation method described in this work is applicable to both type I collagen and type II collagen, these inks have broad potential for various tissue-engineering applications. To the best of our knowledge, this method enabled, for the first time, the 3D-printing of solid scaffolds composed solely of intact, insoluble type II collagen microfibers.

## 2. Materials and Methods

Unless specified otherwise, chemical reagents used were purchased from Sigma-Aldrich (St. Louis, MO, USA).

### 2.1. Preparation of Purified Collagen Fibers

#### 2.1.1. Type I Collagen

Purification of porcine tendon type I collagen fibers used a procedure modified from our previously described method [36]. Briefly, fat and fascia were removed from porcine flexor tendons, and then the tendons were washed in water. The cleaned tendons were then frozen and mechanically comminuted into 0.5 mm slices. At room temperature, the tendon slices were first extracted in purified water to remove water-soluble proteins, followed by 0.5 M HCl in 0.5 M Na_2_SO_4_ for 20–24 h, then in 1 M NaOH in 1 M Na_2_SO_4_ for 1 h followed by 0.3 M NaOH in 0.5 M Na_2_SO_4_ for 20–24 h to remove acidic and basic proteins and some lipids. Extraction in isopropanol twice each for 24 h was then performed to remove the lipid moieties. Finally, the purified porcine type I collagen fibers were air-dried and stored in a cool and dry place until use.

#### 2.1.2. Type II Collagen

Purification of bovine articular cartilage type II collagen fibers was performed using protocols previously described by our group [37,38]. Briefly, bovine articular cartilage shavings from 4 to 6 month-old calves were obtained from a local abattoir (Spear Products Inc., Coopersburg, PA, USA). The shavings were then frozen and mechanically sliced into 0.5 mm slices. Non-collagenous proteins and other contaminants were removed by extracting the cartilage slices at 4 °C, first in 0.2 M NH_4_Cl twice for 20 h each, followed by treatment with 1.0 M NaOH for 1 h then with 0.5 M NaOH for 7 h. After thorough washing with purified water, the slices were extracted in 0.5 M HCl for 24 h, followed by 4% ethylenediaminetetraacetic acid (EDTA) for 20 h, then with 4 M guanidine hydrochloride for 24 h. Finally, the slices were extracted in 99% isopropanol for 24 h and then dried. The resulting purified type II collagen fibers were stored in a cool and dry place until used.

### 2.2. Preparation and Characterization of Collagen Microfibers

Following collagen purification, chemical analyses of different batches of insoluble type I collagen (Supplementary Materials Section S1.1, Table S1) and insoluble type II collagen [38] demonstrated high purity and minimal batch-to-batch variability. Based on these results, purified collagen from 4 independent batches of type I collagen and 3 independent batches of type II collagen were first independently processed (as described below) to generate microfiber size-ranges. Then for each collagen type, corresponding microfiber size-ranges from the independent batches were pooled to obtain sufficient starting material to generate the ink formulations used across experiments.

Microfiber size-ranges were prepared by first cutting purified collagen fibers into small pieces using a Grindomix GM200 mechanical knife mill (Retsch, Haan, NRW, Germany), followed by further size reduction to the micron scale using a SPEX 6775 Freezer/Mill (SPEX Sample Prep, Metuchen, NJ, USA) with the following settings: 10 impacts/second, 2 min per cycle, and 1 min cooldown between cycles. The resulting microfibers were filtered using stainless steel sieves (Cole-Parmer US, Vernon Hills, IL, USA) to obtain several size-ranges: <50 µm, 50–75 µm, 75–100 µm, 100–125 µm, 125–150 µm, 150–175 µm and 175–200 µm. Cryomilling was repeated as needed (up to 10 cycles total) to generate sufficient quantities of each microfiber size-range for subsequent experiments, which were stored in conical tubes with silica gel packs in a cool and dry place until ink preparation.

To investigate the effect of cryomilling on the collagen material’s native macro- and microscopic structure, SDS-PAGE, thermal stability analysis, and light microscope observation were performed.

#### 2.2.1. Light Microscope Analysis

Macroscopic fiber morphology and the inherent swelling behavior of the collagen microfibers were evaluated by first hydrating microfibers at pH 2.5 or pH 7.2 for at least 1 h. The hydrated microfibers were then placed onto glass slides and viewed with a light microscope equipped with a digital color camera (VWR, Radnor, PA, USA).

#### 2.2.2. SDS-PAGE Analysis

SDS-PAGE was performed according to the method of Laemmli [39] to assess the intactness of the collagen α chains following cryomilling of the purified collagen fibers into microfibers. Briefly, 40 mg of sample was first solubilized in 1 mL of pepsin digestion solution (2 mg/mL pepsin in 0.2 M acetic acid). Type I collagen microfibers necessitated overnight digestion at 35 °C, whereas type II collagen microfibers needed only 4 h at room temperature. Following digestion, the samples were spun down and supernatants neutralized with 2 M Tris (Fisher Scientific, Hampton, NH, USA). 60 µL of the neutralized solution was then mixed with 20 µL of 4× Laemmli sample buffer (Bio-Rad Laboratories Inc., Hercules, CA, USA; cat# 1610747) and hydrolyzed in boiling water for 10 min. In total, 5–10 µL of sample and standard protein marker were then loaded into a 4–15% Mini-PROTEAN^®^ TGX pre-cast gel (Bio-Rad Laboratories Inc. cat# 4568083) and subjected to electrophoresis at 100 V at room temperature (Mini-PROTEAN Tetra-Cell, Bio-Rad Laboratories Inc. cat# 1658004). Once completed, the gel was washed in ultrapure water 3 times for 5 min then stained with Bio-Safe™ Coomassie G-250 Stain (Bio-Rad Laboratories Inc. cat# 1610786) for 1 h and then rinsed with ultrapure water until clear bands were seen.

#### 2.2.3. Thermal Stability Analysis

Thermal stability of the purified collagen material before and after cryomilling was evaluated by differential scanning calorimetry (DSC) as an indication of the preservation of native crosslinking. Measurements were performed using a differential scanning calorimeter (Polymer DSC, Mettler Toledo, Columbus, OH, USA) at a heating rate of 5 °C/min over a temperature range of 30–100 °C. Samples (~3 mg dry weight) were hydrated with 20 μL PBS (Thermo Fisher Scientific, Waltham, MA, USA) and hermetically sealed in aluminum pans. Denaturation temperature (Td, °C) was determined with Mettler Toledo STAR^e^ software (ver 14.00) as the peak temperature of the thermogram. Average Td values were obtained by analyzing microfibers from each of the independent collagen isolations prior to pooling corresponding size-ranges into a single source material as described above.

### 2.3. Preparation of Collagen Microfiber-Based Inks

Size-ranges of collagen microfibers from the single pooled collagen source material, obtained from independent collagen isolations as described above, were used to prepare collagen microfiber-based inks at three pH conditions: the isoelectric point (pH4.6 for type I collagen and pH5.0 for type II collagen) (sodium acetate buffer), pH 7.2 (phosphate-buffered saline) (Thermo Fisher Scientific), and pH 2.5 (0.05 M lactic acid).

**Figure 1:**
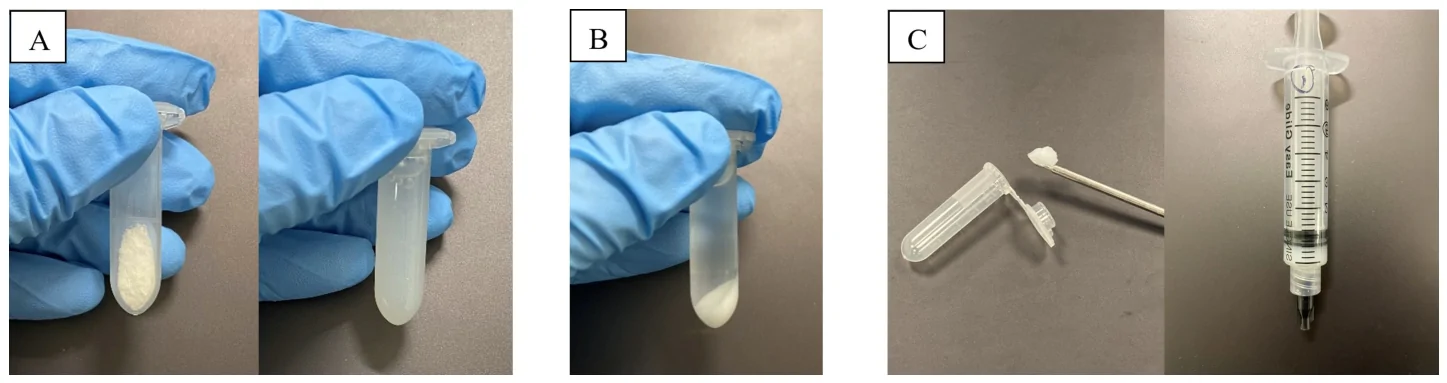
Preparation of collagen microfiber-based inks. (**A**) Microfibers (**left**) hydrated in buffer solution for 1–3 h (**right**). (**B**) Centrifugation to pellet microfibers. (**C**) Excess buffer removed, and the pelleted, hydrated microfibers transferred to a syringe for extrusion.

The general procedure for preparing collagen microfiber-based inks at room temperature is shown in Figure 1 using type I collagen microfibers hydrated at pH 4.6 as an example. This procedure is applicable for any pH in combination with any microfiber size-range for either type I collagen or type II collagen:

A size-range of purified type I collagen microfibers was first hydrated in sodium acetate buffer (pH 4.6) at a ratio of 10 mg/mL with constant mixing on a Fisherbrand™ nutating mixer (Fisher Scientific). Once equilibrium hydration was achieved (generally 1–3 h depending on the microfiber size-range) excess buffer was removed via centrifugation at 14,000× *g* for 6 min (Centrifuge 5415D, Eppendorf, Hamburg, Germany). The supernatant was then carefully removed, and any residual on the tube walls were blotted with lint-free Kimtech™ Kimwipes (Kimberly-Clark, Irving, TX, USA). The residual buffer volume was calculated by subtracting the volume of buffer removed after centrifuging from the initial volume of buffer. This volume plus the volume of collagen (taking the density of collagen to be 1.41 g/mL [40]) was then used to determine the concentration (%(*w*/*v*)) of the collagen in the resulting pellet. The pelleted hydrated microfibers were then loaded into a syringe to be used as the ink, and then briefly centrifuged to remove macroscopic air bubbles introduced during the transfer step.

### 2.4. Printability of Collagen Microfiber-Based Inks

#### 2.4.1. Nozzle Size

Type I collagen and type II collagen microfiber-based inks were prepared as described above and printed at room temperature onto 100 mm plastic Petri dishes using a mechanical syringe extrusion-based printer (Engine SR, Hyrel3D, Norcross, GA, USA) with both the print bed and extrusion head at room temperature. Two-layer crosshatch patterns (10 mm × 10 mm) were initially printed using 22G, 19G, or 16G blunt-tip needles 1–3 cm long (McMaster-Carr, Elmhurst, IL, USA) to evaluate nozzle-size suitability and filament quality for each ink formulation.

#### 2.4.2. Construct Integrity

To properly illustrate the influence of collagen density on construct stability, only type I collagen microfibers were utilized because a broader range of ink densities could be prepared using type I collagen microfibers compared to type II collagen microfibers. Crosshatch patterns, as described above, were first printed for 4 layers to observe construct integrity immediately after printing without any post-printing processing (freeze-drying and crosslinking). Finally, 8-layer crosshatch patterns (~9 mm in height) were printed using a 16G blunt-tipped nozzle and inks of increasing collagen density. Changes in height of these 8-layer constructs were recorded after a period of 2 h at room temperature without post-printing processing (freeze-drying and crosslinking).

### 2.5. Post-Printing Processing and Scaffold Characterization

#### 2.5.1. Freeze-Drying

Prints were immediately frozen at −20 °C for 1 h prior to lyophilization (FreeZone 2.5L, Labconco, Kansas City, MO, USA).

#### 2.5.2. Crosslinking

As the inks are collagen-based, two common collagen crosslinking methods (formaldehyde and EDC/NHS) were employed as examples for post-printing processing. Other collagen crosslinking methods that have not been particularly specified in this work may also be applicable for post-printing crosslinking [41,42,43].

Scaffolds for mechanical testing were crosslinked with formaldehyde vapor, though crosslinking with other low molecular weight aldehyde compounds can also be used (e.g., glycolaldehyde and glutaraldehyde). The formaldehyde vapor was generated from a 1% formaldehyde solution (Thermo Fisher Scientific) at room temperature in a custom-made enclosed chamber equipped with fans to circulate the vapor. Formaldehyde residuals were then removed via washing with 0.1 M Tris buffer (Fisher Scientific) for 5 days followed by purified water for 1 day to remove Tris residuals. All washing steps were performed at 37 °C under constant shaking and replacing solutions twice daily. The washed scaffolds were then lyophilized and stored in a cool and dry place until use.

Scaffolds for in vitro cell culture were crosslinked in 1 mL of EDC/NHS solution (70% ethanol (Fisher Scientific) containing 9 mM 1-ethyl-3-(3-dimethylaminopropyl)carbodiimide (EDC) (Thermo Fisher Scientific), 3.6 mM N-Hydroxysuccinimide (NHS) (Thermo Fisher Scientific) and 50 mM 2-morpholinoethanesulfonic acid monohydrate (MES) (Thermo Fisher Scientific)) per 3 mg of collagen scaffold for 5 h at room temperature. After crosslinking, scaffolds were washed in 0.1 M sodium phosphate dibasic and purified water, then freeze-dried overnight. Prior to cell culture, scaffolds were sterilized using ethylene oxide (Anprolene AN74i, Andersen Sterilizers, Haw River, NC, USA).

#### 2.5.3. SEM Analysis

To properly assess the effect of collagen density on cross-sectional pore sizes of printed constructs, only type I collagen microfibers were utilized since a wider range of densities were able to be obtained using type I collagen microfibers compared to type II collagen microfibers. Detail of the cross-sections and surface morphologies of lyophilized, printed constructs were viewed via SEM (Hitachi S4800, Hitachi Ltd., Tokyo, Japan) at an acceleration voltage of 5 kV after mounting samples on carbon tape and coating with a thin layer of iridium. Pore sizes were measured via ImageJ (ver 1.53m) defined as the length of the longest axis.

#### 2.5.4. Effect of Collagen Microfiber Size and Density on Mechanical Properties

**Figure 2:**
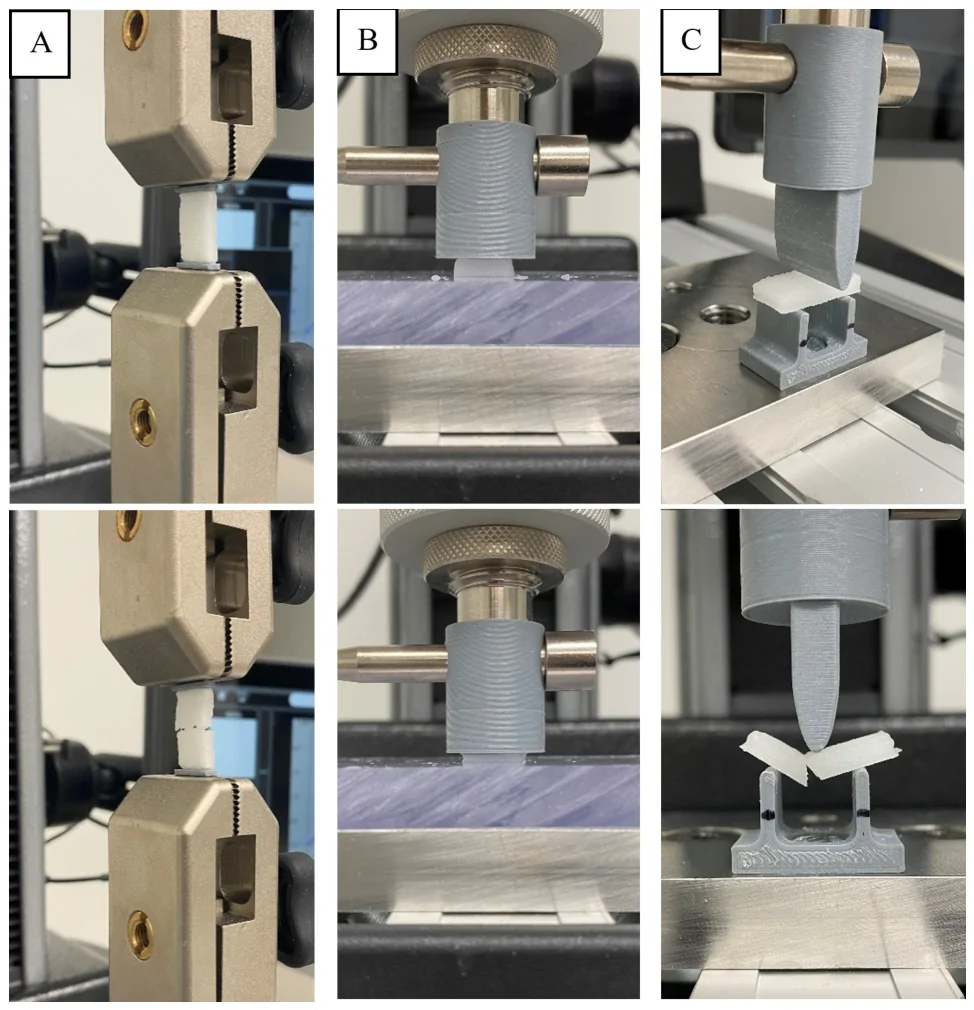
Mechanical testing setups. (**A**) Tensile, (**B**) unconfined compression, and (**C**) 3-point bending tests with samples at start of test (**top**) and end of test (**bottom**).

To properly assess the effects of microfiber size and density on the mechanical properties of fabricated scaffolds, only type I collagen microfibers were utilized since a wider range of densities were able to be obtained using type I collagen microfibers compared to type II collagen microfibers. Tensile, unconfined compression, and three-point bending tests (Figure 2) were performed on solid block scaffolds (*n* = 4) printed using either ink formulations with a fixed collagen density of 10% and different microfiber size-ranges, or ink formulations with a fixed microfiber size-range and varying collagen densities (approximately 2.6%, 13%, and 20% respectively). Additional details regarding ink formulations (including hydration pH and printing parameters) can be found in Supplementary Materials Section S1.2.

Scaffolds were first hydrated in PBS at room temperature for 30 min. Vacuum was initially applied to remove interior air bubbles. After hydrating, samples were gently blotted with lint-free Kimwipes to remove excess PBS before testing. All mechanical tests were performed at room temperature using a Chatillon CS2–225 mechanical tester (AMETEK, Berwyn, PA, USA) at a crosshead speed of 1 mm/min.

For tensile testing (Figure 2A), rectangular scaffolds (2 cm × 1 cm × 0.3 cm; Length × Width × Height; L × W × H) were first glue bonded to custom-designed tabs prior to rehydration. Samples were then clamped along their long axis using the tabs to prevent direct contact between the samples and the clamps, minimizing sample slippage during testing. Samples were then loaded under uniaxial tension along their long axis until failure.

For unconfined compression testing (Figure 2B), square scaffolds (1 cm × 1 cm × 0.3 cm; L × W × H) were placed between impermeable plates. A uniaxial compressive load was then applied to the square face of the sample through the upper plate after a preload of 0.05 N was met to ensure even contact between the sample and the plate. Samples were then compressed to 50% of their original thickness (50% strain).

For 3-point bending testing (Figure 2C), rectangular scaffolds (2 cm × 1 cm × 0.3 cm; L × W × H) were placed evenly across two supports spaced 13 mm apart. The supports had rounded tips approximately 1 mm in diameter, and the loading tip to apply the force had a round edge approximately 1 mm in diameter. Prior to performing the test, a preload of 0.05 N was applied to ensure even contact across the whole width of the loading tip and the sample. Once the preload was met, a compressive load was then applied across the center width of the sample until failure or until the sample sides bent beyond 45°. Flexural stress (σ*_f_*) and strain (ε*_f_*) were calculated from Equations (1) and (2) respectively, below, where *F* is the force recorded by the mechanical tester, *S* is the distance between supports, *W* is the width of the sample, *H* is the sample thickness, and *D* is the crosshead displacement (i.e., sample deflection) [44].

(1) $$\sigma_{f}=\frac{3(F)(S)}{2(W)(H^{2})}$$

(2) $$\varepsilon_{f}=\frac{6(D)(H)}{S^{2}}$$

Except for 3-point bending testing, stress was calculated by dividing the applied force by the sample cross-sectional area, and strain was calculated by dividing the crosshead displacement by the initial sample length along the testing axis (crosshead position was zeroed once preload was met as described above). Modulus was determined by the slope of the initial linear portion of the stress–strain curve following the toe region. Toe regions were generally from 0 to 5% strain. Representative stress–strain curves were generated for each group by averaging stress and strain data up to the ultimate stress from each sample.

### 2.6. In Vitro Cell Compatibility of High-Density Collagen Scaffolds

The ability of high-density type I collagen microfiber-based scaffolds to support cell proliferation was evaluated using solid blocks (*n* = 4) with dimensions of 1 cm × 1 cm × 0.4 cm (L × W × H). Scaffolds were prepared using 11% and 20% type I collagen microfiber-based inks printed in a crosshatch pattern with no gaps between filaments. Additional details regarding ink formulations and scaffold fabrication are provided in Supplementary Materials Section S1.4. Scaffolds were inoculated with 2.5 × 10^5^ L929 fibroblasts (Sigma-Aldrich) via injection into the center of the scaffolds and cultured statically for 7 days in growth medium (DMEM containing 10% FBS and 1% penicillin–streptomycin (Thermo Fisher Scientific)) in a humidified incubator at 37 °C and 5% CO_2_ (CO_2_ Incubator Basic, VWR). Cell proliferation was analyzed via AlamarBlue (Thermo Fisher Scientific) following manufacturer’s instructions and DAPI staining (Thermo Fisher Scientific) of 10 µm thick cryosections.

The ability of high-density type II collagen microfiber-based scaffolds to support chondrocyte redifferentiation was evaluated using 3 mm thick discs (*n* = 6) cut from cylinders prepared using a 15% type II collagen microfiber-based ink. Cylinders were designed in CAD software (AutoDesk^®^ Inventor^®^ Professional 2022 ver 26.0.15300.0000) with dimensions 5Ø × 10 mm height and converted into a STL file for 3D-printing. An additional cylinder was prepared by manual injection-molding the same ink into a 96-well plate, followed by freeze-drying and crosslinking in the same manner as the 3D-printed cylinder to determine whether extrusion method affected pore structure. Histological methods used to observe pore structure are provided in Supplementary Materials Section S2.1. Bovine chondrocyte redifferentiation was performed using a method modified from our previously described protocol [38]. Briefly, scaffolds were inoculated via injection with 1 × 10^6^ passage 1 (P1) bovine chondrocytes isolated from 4.5-month-old calves. After allowing cells to attach overnight in growth medium under static conditions in a standard 37 °C and 5% CO_2_ incubator, the cell-laden scaffolds were cultured for an additional 7 days under 100 RPM on an orbital shaker (Fisher Scientific). Scaffolds were then transferred to a hypoxic incubator (5% O_2_) (Thermo Forma 3130, Thermo Fisher Scientific) and cultured in chondrocyte differentiation medium (CDM) [38] under the same orbital shaking conditions. Scaffolds were harvested after 2 and 4 weeks of culture in CDM for histological analyses of glycosaminoglycan deposition (GAG) using Toluidine Blue staining, and gene expression analysis of *Col1a1*, *Col2a1*, *ACAN*, and the house-keeping gene *GAPDH* using qRT-PCR. Additional details regarding scaffold fabrication, chondrocyte isolation and expansion, medium formulations, and qRT-PCR can be found in Supplementary Materials Sections S2.1–S2.4 and our previous report [38].

### 2.7. Potential Applications of High-Density Collagen Inks

#### 2.7.1. Bone Mineral Composite Ink for Bone Regeneration

Carbonate apatite was obtained from the cancellous bone of cleaned bovine femoral heads by grinding the bone down into a powder and then heating the powder at approximately 500 °C for 24 h. The mineral particles that remained were further ground and sieved to collect particles 30–45 µm in size.

To incorporate the bone mineral particles into an ink, a 10% type I collagen microfiber-based ink was first prepared. The syringe containing the ink was then connected via a leur-lock to leur-lock connecter to a second syringe containing the mineral particles. The amount of bone mineral added was weighed out to achieve final mineral-to-collagen ratios of either 60:40 or 80:20. The mineral was then incorporated into the ink by first passing the collagen ink into the syringe containing the mineral, and then repeatedly transferring the mixture back and forth between the two syringes. The syringe was then briefly centrifuged to remove any air bubbles introduced by the mixing.

#### 2.7.2. Tube Fabrication

A custom setup for high-throughput fabrication of hollow tubes was built by first attaching a polypropylene rod to a spinning motor and then mounting the assembly onto the print bed of our Hyrel 3D ESR printer. The rod was positioned such that inks can be extruded directly along the length of the rotating rod to produce hollow tubes after lyophilization.

### 2.8. Statistical Analysis

All quantitative data were presented as mean ± standard deviation (SD). Statistical comparisons between two groups were performed using unpaired two-tailed Student’s *t*-test. A *p*-value of less than 0.05 was considered statistically significant. Sample sizes (*n*) for each group are indicated in the figure legends. Graphs were created with Microsoft Excel.

## 3. Results

### 3.1. Preparation and Characterization of the Collagen Microfibers

**Figure 3:**
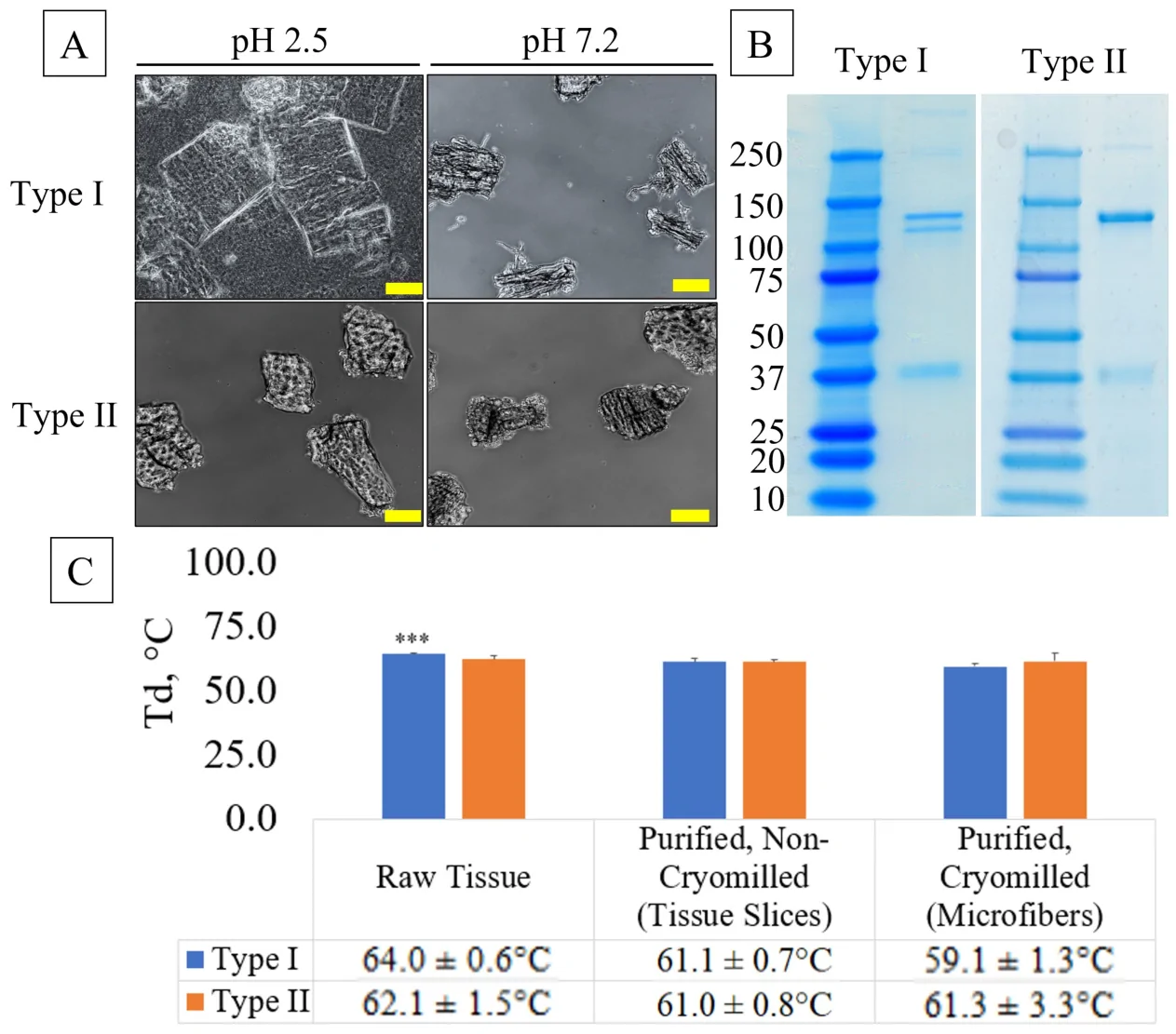
Characterization of the collagen microfibers. (**A**) Light microscope images of purified type I collagen and type II collagen microfibers (125–150 µm in size) hydrated at different pH values. Scale bars: 100 µm. (**B**) SDS-PAGE analysis of the purified type I collagen and type II collagen microfibers shows bands corresponding to the α1(I) and α2(I) chains of type I collagen (~120–140 kDa) and a single major band corresponding to the α1(II) chains of type II collagen (~140 kDa). The band at 37 kDa corresponds to pepsin. Faint γ and β bands are also visible above and at 250 kDa respectively. (**C**) Average denaturation temperatures (Td, °C) of the purified type I collagen (*n* = 4) and type II collagen (*n* = 3) materials before and after cryomilling compared to their native tissues (tendon and articular cartilage, respectively, *** *p* < 0.001, *t*-test).

Light microscopic images of type I collagen and type II collagen microfibers under different pH conditions are shown in Figure 3A. Despite undergoing 10 cycles of cryomilling, type I collagen microfibers retained the inherent swelling behavior characteristic of intact, insoluble type I collagen fibers at pH 2.5, as evidenced by their transparency due to up-taking solution. At pH 7.2, the fiber structure for both collagen types remained easily observable, indicating that their gross morphology was not disrupted by the cryomilling process. For the type II collagen microfibers at pH 2.5, swelling was not observed to the same extent as for the type I collagen microfibers, and their appearance was similar to the type II collagen microfibers at pH 7.2. This observation is consistent with previously reported characteristics of purified, insoluble type II collagen [6].

The preservation of the collagen triple-helix structure after cryomilling was demonstrated via SDS-PAGE analysis (Figure 3B). The α1(I) and α2(I) chains of type I collagen (~120 and ~140 kDa respectively) and the α1(II) chains of type II collagen (single major band at ~140 kDa) migrated at positions corresponding to the intact α chains of both collagen types without showing extra distinguishable smears or lower molecular weight bands (other than pepsin at ~37 kDa). This suggests that cryomilling did not result in detectable damage to the collagen’s primary structure.

DSC analysis (Figure 3C) shows that the average thermal stability temperatures of the purified collagen materials were slightly lower compared to their corresponding native tissues (porcine flexor tendon and bovine articular cartilage). More importantly, the average thermal stability temperatures of the type I collagen and type II collagen microfibers were similar to the purified materials prior to cryomilling. This indicates that native crosslinks preserved after undergoing the purification process were retained after cryomilling, thus preserving native tissue collagen organization of the purified materials.

Overall, these data demonstrate the successful size reduction of collagen fibers into microfibers via cryomilling, without denaturation or significant disruption of the native collagen structure.

### 3.2. Preparation and Printability of the Collagen Microfiber-Based Inks

#### 3.2.1. Obtainable Densities

**Figure 4:**
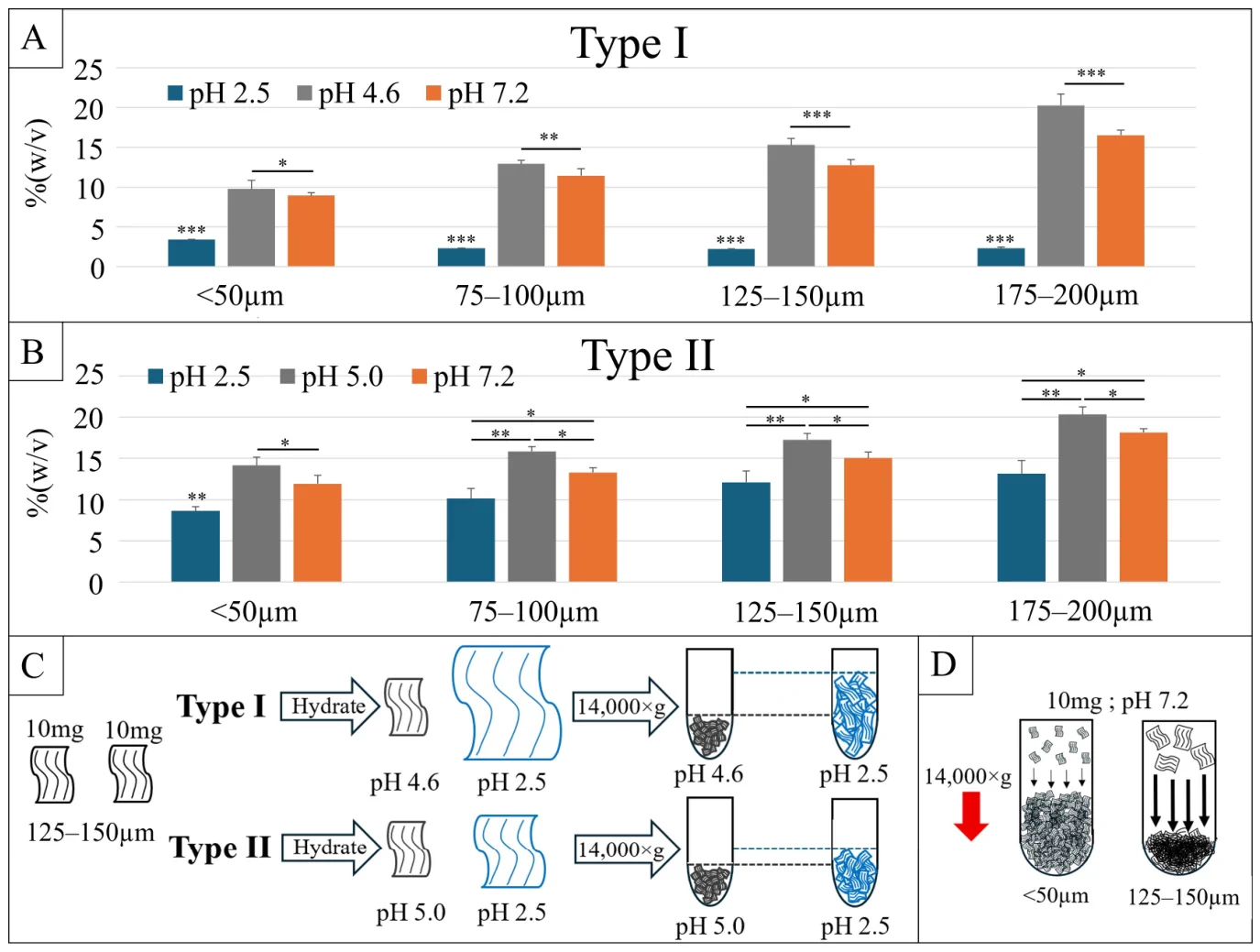
Achievable average densities for collagen microfiber-based inks and effects of microfiber swelling and size. Densities for (**A**) type I collagen and (**B**) type II collagen microfiber size-ranges hydrated at pH 2.5, at their isoelectric point (IP), pH 4.6 or pH 5.0, and at pH 7.2, following centrifugation at 14,000× *g* for 6 min at room temperature (* *p* < 0.05, ** *p* < 0.01, *** *p* < 0.001, *n* = 4, *t*-test). Schematic representations of the effects of (**C**) microfiber swelling and (**D**) microfiber size on ink density following centrifuging. Differences in sedimentation velocity of different microfiber sizes are indicated by the magnitude of the black arrows in (**D**).

Achievable average densities of collagen microfiber-based inks are shown in Figure 4. While using identical centrifugation parameters (14,000× *g* for 6 min), inks prepared from microfibers of the same size-range but hydrated at different pH conditions exhibited the highest average densities at the collagen’s isoelectric point and the lowest average densities at pH 2.5. By leveraging the polyelectrolytic nature of collagen, microfiber packing when preparing the ink can be controlled by modulating the degree of swelling of microfibers through pH adjustment. In other words, greater microfiber swelling results in reduced packing efficiency compared to the more compact microfiber geometry when hydrated at the isoelectric point (Figure 4C). Interestingly, at a fixed pH, increasing microfiber size resulted in increasing achievable average densities (Figure 4A,B), implying that larger microfibers would be more readily compacted by centrifugation forces resulting in greater sedimentation as shown in Figure 4D.

Since type II collagen microfibers do not exhibit swelling to the same extent as type I collagen microfibers, the influence of pH on achievable average densities was less pronounced. For example, there was only an approximately 1.4-fold difference between achievable average densities at pH 2.5 and pH 5.0 (IP) using type II collagen microfibers 125–150 µm in size compared to an approximately 6.9-fold difference observed if type I collagen microfibers were used under the same conditions (IP is pH 4.6) (Figure 4). Therefore, the range of achievable average densities when preparing type II collagen microfiber-based inks was generally narrower compared to type I collagen microfiber-based inks.

Overall, the average collagen content of type I collagen microfiber-based inks was able to be adjusted from 2% (20 mg/mL) to 20% (200 mg/mL) by controlling microfiber swelling through pH adjustment. When preparing type II collagen microfiber-based inks, achievable average densities below 8% (80 mg/mL) were not attainable due to type II collagen’s inability to swell to the same extent as type I collagen.

The observed variability when preparing multiple tubes of the same ink formulation likely resulted from the use of a range of microfiber sizes instead of a consistent size (e.g., 75–100 µm in size instead of just 100 µm). Even within a controlled size-range, variations in microfiber size distribution may influence the amount of buffer remaining after centrifuging, as different size microfibers can hold different amounts of buffer once hydrated. However, because each microfiber size-range was limited to a narrow range of 25 µm through sieving, batch-to-batch ink variability remained within 1% density of the achievable average density for each ink formulation based on standard deviations as shown in Figure 4A,B. This result demonstrates that the microfiber ink preparation method provides consistent and reproducible control over collagen densities.

#### 3.2.2. Printability Assessment: Nozzle Size and Construct Integrity

**Figure 5:**
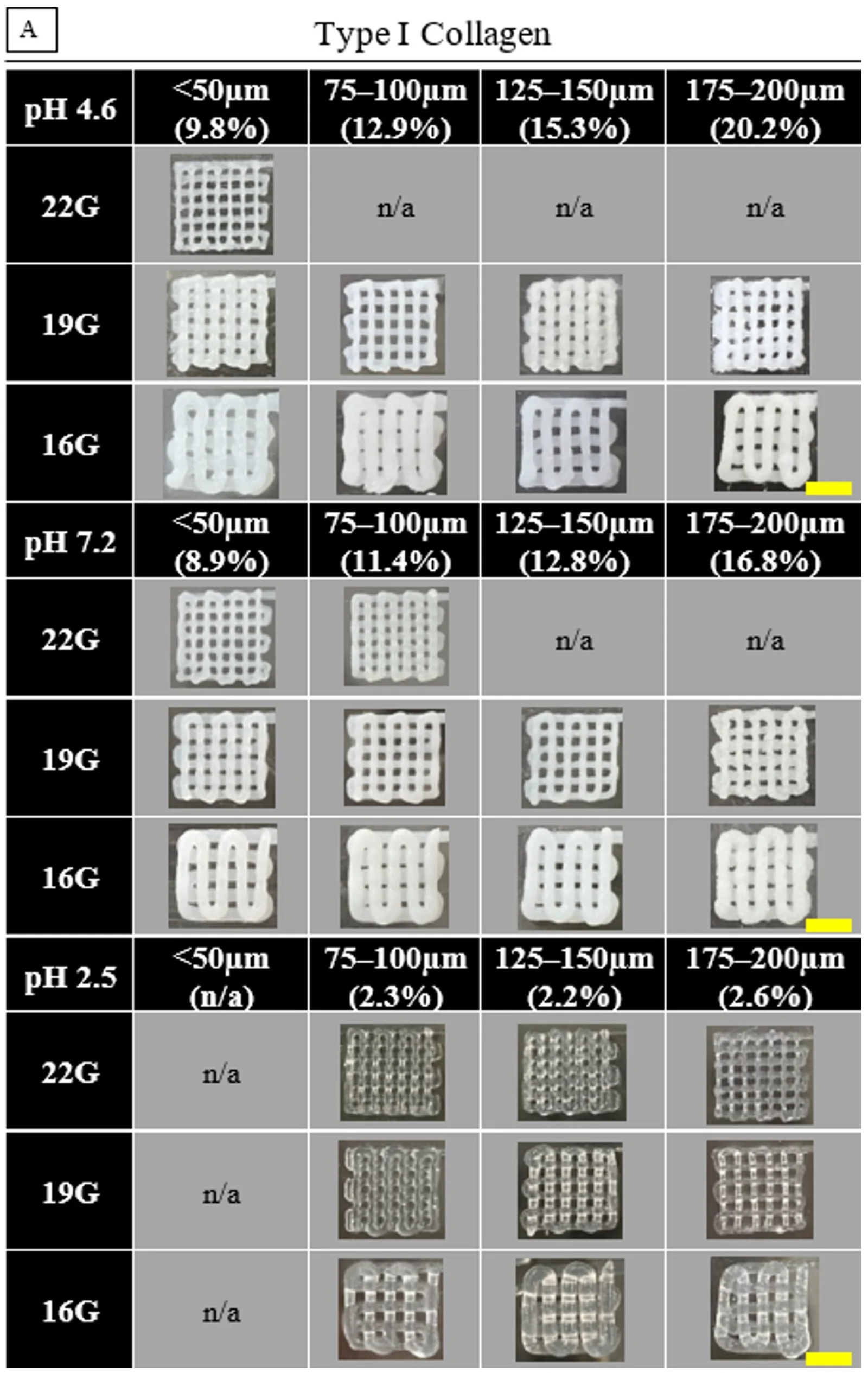

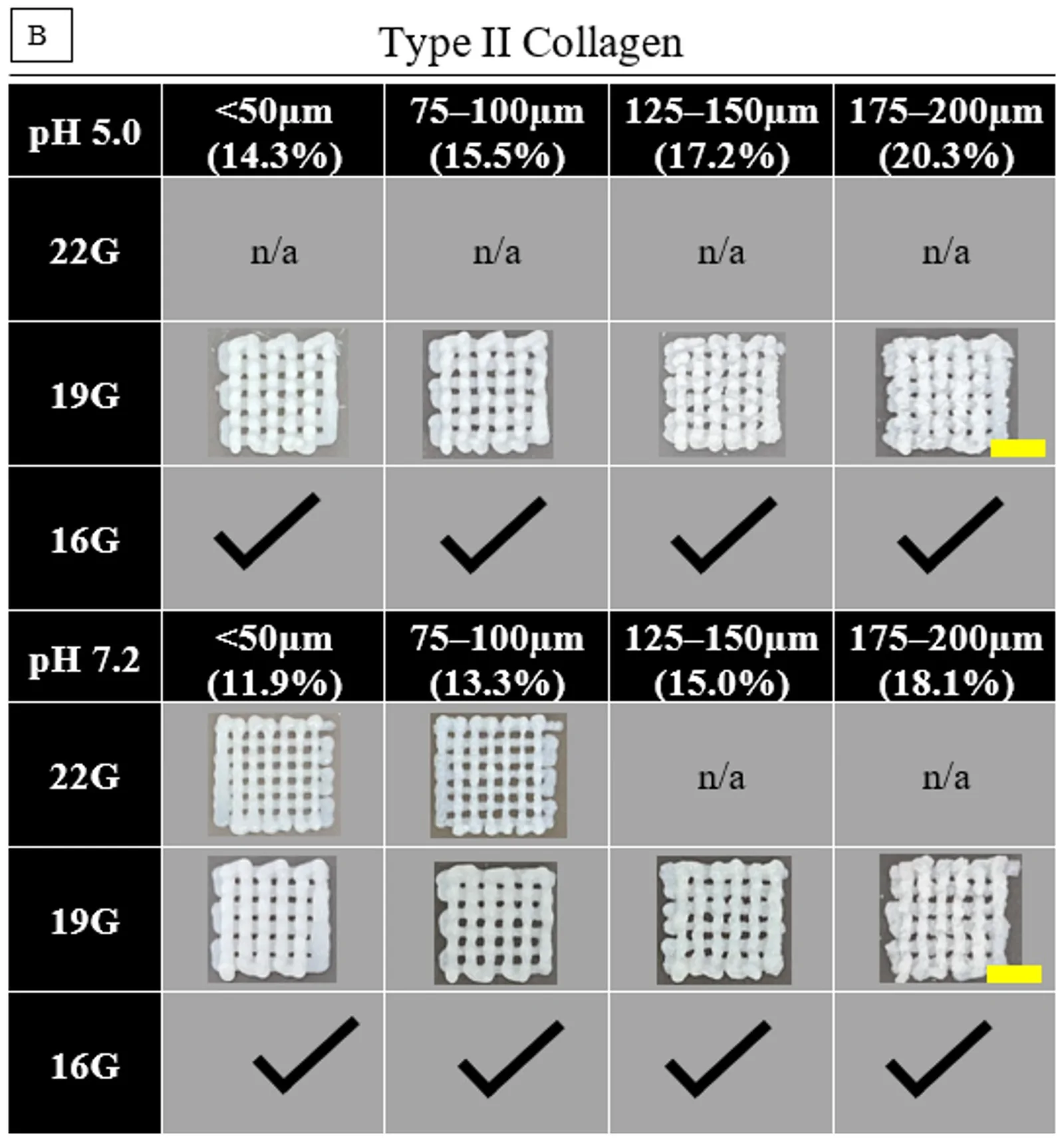
Nozzle size printability assessment. Top-view images of 2-layer crosshatch patterns immediately after printing (**A**) type I collagen microfiber-based inks and (**B**) type II collagen microfiber-based inks with varying microfiber size-ranges hydrated at different pH, and centrifuged at 14,000× *g* for 6 min. Based on the assessment for type I collagen microfiber-based inks, the type II collagen microfiber-based inks were considered printable with the 16G nozzle when printing with the 19G nozzle was successful (as indicated by the checkmarks). The average percent collagen density is included in parentheses. Note the progressively grainier filament texture as ink density increases. Unsuccessful printing is indicated by “n/a”. Scale bars: 5 mm.

Figure 5A shows the effect of nozzle diameter for room-temperature printing. Microfiber size-ranges of various densities were able to pass through nozzles when sufficiently large diameters were used, as observed with the 19G and 16G nozzles. These successfully extruded inks exhibited good filament shape fidelity, with average filament spreading ratios ranging from 0.88 to 1.14, where a value of 1 indicates filament width matches nozzle diameter. The printed crosshatch patterns also demonstrated acceptable pore shape fidelity, with average values ranging from 0.84 to 1.2 (values closer to 1 indicate an ideal square shape) (Supplementary Materials Section S3.1, Table S4) [45]. However, fully closed pores were evident when printing low-collagen inks prepared at pH 2.5 using the 16G nozzle (Figure 5A). Partial or complete clogging occurred only with the smallest nozzle tested (22G), characterized by intermittent or burst extrusions, extrusion of buffer without collagen leading to ink dehydration, or complete blockage with no material output. Otherwise, printing failed when the ink formulation could not form stable filaments, which occurred only for the type I collagen microfibers < 50 µm in size hydrated at pH 2.5. To elucidate the underlying cause, rheological properties of this non-printable, low-density ink formulation (including G′, the elastic modulus, and G″, the viscous modulus) were compared with those of another low-density ink formulation that successfully produced stable filaments (175–200 µm microfibers hydrated at pH 2.5) (Supplementary Materials Section S3.2, Figure S3A,B).

Although both formulations exhibited predominantly elastic, solid-like behavior during a frequency sweep (G′ > G″ across the tested frequency range), the <50 µm microfiber formulation showed considerably lower G′ values of approximately 160–180 Pa at low strains during a strain sweep compared to the 175–200 µm microfiber formulation, which exhibited values of approximately 800–900 Pa at low strains. Consistent with the strain sweep, the 175–200 µm microfiber formulation also exhibited substantially higher G′ values across the tested frequency range, indicating that the low-density ink consisting of the 175–200 µm microfibers exhibited greater elastic stiffness and resistance to deformation than the low-density ink consisting of the smaller < 50 µm microfibers. These findings suggest that, for the low-density (2–3%) formulations prepared at pH 2.5, elastic strength may depend on microfiber size, as larger hydrated microfibers may form a more robust particle network. It follows then that a sufficiently large microfiber size following hydration at pH 2.5 may be important for filaments of the corresponding ink formulations to resist deformation and retain their shape. Therefore, the lower elastic strength of the <50 µm microfiber formulation may stem from the smaller microfibers forming a less elastic bulk ink network than the larger microfibers, which likely contributed to the formulation’s greater susceptibility to filament collapse following extrusion.

Since all type I collagen microfiber-based ink formulations were able to extrude through the 19G nozzle (Figure 5A), the printing demonstration for type II collagen microfiber-based inks (Figure 5B) did not include the 16G nozzle, as inks that extruded through the 19G nozzle were assumed to also extrude through the larger 16G nozzle. Type II collagen microfiber-based inks prepared at pH 5.0 and 7.2 were selected for this demonstration because densities lower than 5% (50 mg/mL) could not be achieved through swelling type II collagen microfibers at pH 2.5, unlike the type I collagen microfibers. Similarly to type I collagen microfiber-based inks, all formulations were successfully extruded through the 19G nozzle and partial or complete clogging occurred only when the smallest nozzle (22G) was used, resulting in intermittent extrusion, burst extrusion, or complete blockage. Following initial printing (Figure 5B), the type II collagen microfiber-based inks also exhibited acceptable filament and pore shape fidelity, with average filament spreading ratios ranging from 1.06 to 1.18 and average pore shape fidelity values ranging from 0.80 to 1.01 (Supplementary Materials Section S3.1, Table S4).

Unlike type I collagen microfiber-based inks, the type II collagen microfiber-based inks with collagen densities exceeding 15% exhibited filament breakage when second-layer filaments were deposited over gaps between first-layer filaments. This observation may reflect differences in hydration behavior between the two collagen types, as ink cohesiveness may also depend on the collagen’s ability to retain water within deposited filaments. To investigate this possibility, the rheological properties of type I collagen and type II collagen microfiber-based inks were compared using formulations prepared at their isoelectric points (pH 4.6 or pH 5.0, respectively) with the same microfiber size-range and collagen density (Supplementary Materials Section S3.2, Figure S3C,D). At low strains during a strain sweep, even though the type II collagen formulation exhibited a higher G′, the type I collagen formulation exhibited a higher yield strain. In contrast, the type I collagen formulation during a frequency sweep exhibited a higher G′ across the tested frequency range, suggesting greater elastic strength over a broader range of deformation timescales. Together, these results suggest that the type I collagen formulation can produce filaments that accommodate greater deformation than the type II collagen formulation, which exhibited a stiffer, less deformation-tolerant response consistent with its observed brittle-like behavior despite both formulations having the same microfiber size-range and collagen density. Since type II collagen does not swell to the same extent as type I collagen, the hydrated type II collagen microfibers may retain a more discrete, particle-like structure in the ink, resulting in filaments with a greater susceptibility to fracture at densities greater than 15% as observed in Figure 5B. The greater swelling of the type I collagen microfibers, on the other hand, may promote a more compliant and therefore cohesive filament structure that better accommodates mechanical stresses encountered when spanning gaps.

Because type I collagen microfibers enabled a broader range of achievable ink densities, multilayer construct integrity was further evaluated using these formulations. The printable ink formulations in Figure 5A were extruded into the same crosshatch pattern, initially for 4 layers, using the same selection of nozzle sizes (Supplementary Materials Section S3.3, Figure S4). Immediately after printing, open pores were observed with no significant sagging across the constructs. However, greater pore closure was observed in the peripheral pores when using the largest nozzle diameter (16G). The large diameter of the nozzle resulted in curved filament deposition during 90-degree turns, ultimately affecting the intended structure as the number of layers increased. For ink formulations prepared at pH 4.6 and pH 7.2, printing with nozzle diameters smaller than 16G generally resulted in better preservation of peripheral pores (Supplementary Materials Section S3.4, Figure S5A–C). In contrast, for ink formulations prepared at pH 2.5, the 22G nozzle better preserved peripheral pores only for the 175–200 µm microfiber size-range. This may be attributed to the lower filament spreading ratio of this ink formulation compared with the other microfiber size-ranges hydrated at pH 2.5 that were also printed using the 22G nozzle (Supplementary Materials Section S3.1, Table S4). Filaments extruded from the low-density collagen microfiber ink formulations prepared at pH 2.5 also exhibited a greater tendency to fuse during printing than those produced from higher-density collagen formulations. When comparing two formulations consisting of the same microfiber size-range but differing in collagen densities, the low-density formulation exhibited consistently lower G′ values during both frequency and strain sweeps, as well as lower yield strain and G′/G″ crossover strain values during the strain sweep (Supplementary Materials Section S3.2, Figure S3E,F). These results demonstrate that increasing collagen density enhances the mechanical properties of the ink as expected. The greater tendency of filaments to fuse may therefore stem from reduced mechanical properties associated with lower collagen density. This may have contributed to variations in construct height when the same nozzle was used across different low-density collagen microfiber ink formulations, as well as reduced filament definition observed with the 16G nozzle following freeze-drying (Supplementary Materials Section S3.3, Figure S4).

**Figure 6:**
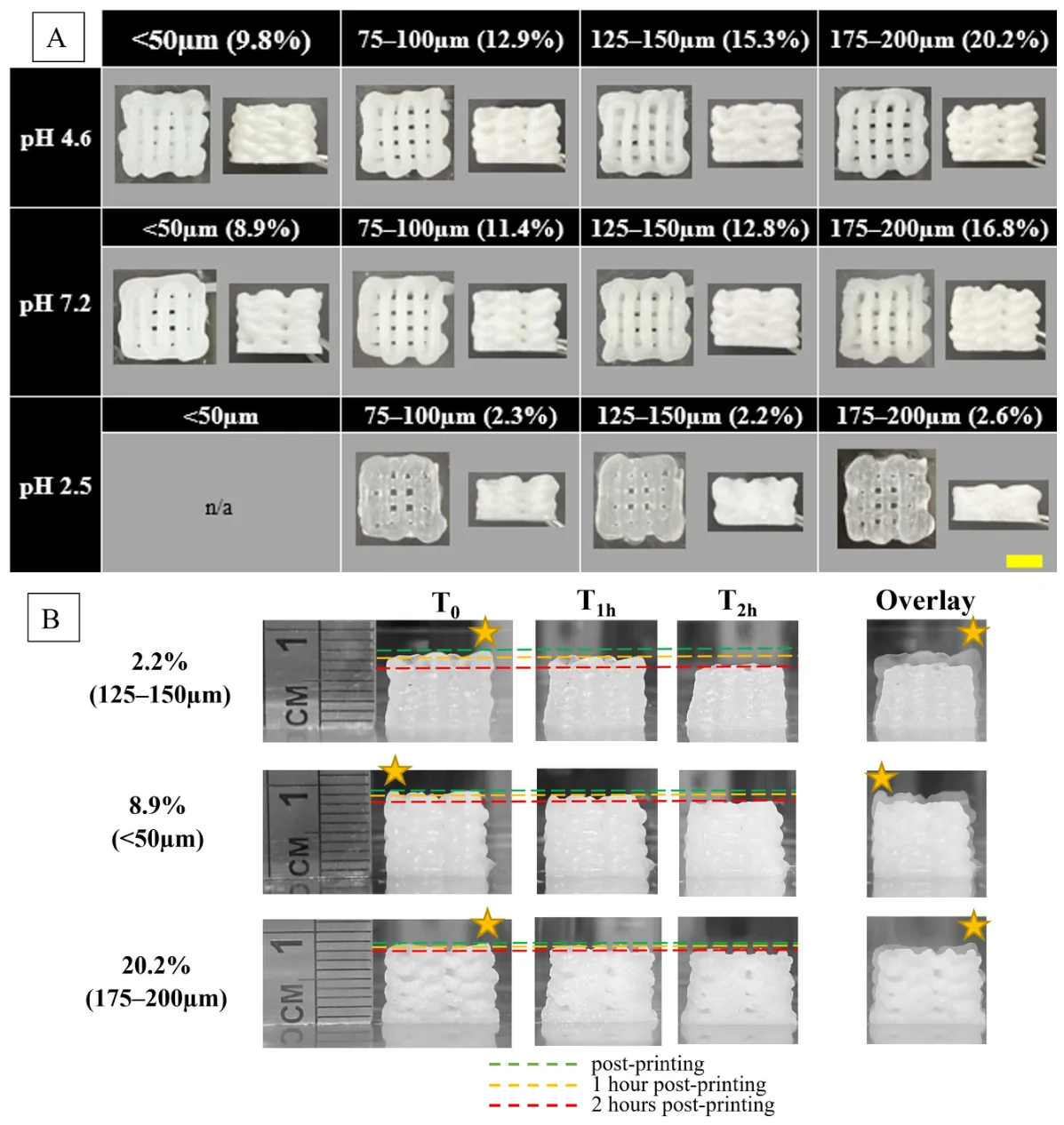
Construct integrity directly after printing. (**A**) Top view immediately post-printing and side view after lyophilization of crosshatch patterns consisting of 8 layers of ink extruded through a 16G nozzle. Average percent collagen content is included in parentheses. Non-printable ink formulations are indicated by “n/a”. Scale bar: 5 mm. (**B**) Stability at room temperature over time of non-crosslinked, 8-layer constructs with increasing collagen content. The yellow star indicates the reference point used for height comparisons. An overlay comparison of the constructs at T_0_ and T_2h_ are also shown.

Finally, 8-layer crosshatch patterns were printed with type I collagen microfiber-based inks using a 16G nozzle to evaluate the largest constructs achievable using the nozzle sizes in this study (Figure 6A). Top-view images revealed greater pore closure than what was observed for the 4-layer constructs, especially for peripheral pores. This is likely due to the increased weight of the filaments combined with the lower printing resolution associated with the 16G nozzle. Pore area reduction analysis confirmed this trend (Supplementary Materials Section S3.4, Figure S5D). Nevertheless, printed constructs immediately after printing maintained sufficient structural stability without severe collapse or overall structural deformation. Consistent with observations for the 4-layer constructs, the low-density ink formulations exhibited greater variability in construct height and reduced filament definition after freeze-drying compared with the higher-density formulations.

The structural stability of these 8-layer constructs was further evaluated using the stability assay shown in Figure 6B. Freshly printed 8-layer crosshatch patterns with varying type I collagen microfiber densities were monitored at room temperature, and changes in construct height were recorded. Height loss decreased with increasing collagen density, consistent with the lower water content of the higher-density ink formulations since deformation in this case was likely driven by water evaporation as the constructs dried out, which reduced filament volume.

### 3.3. Post-Printing Processing and Scaffold Characterization

#### 3.3.1. SEM Analysis

**Figure 7:**
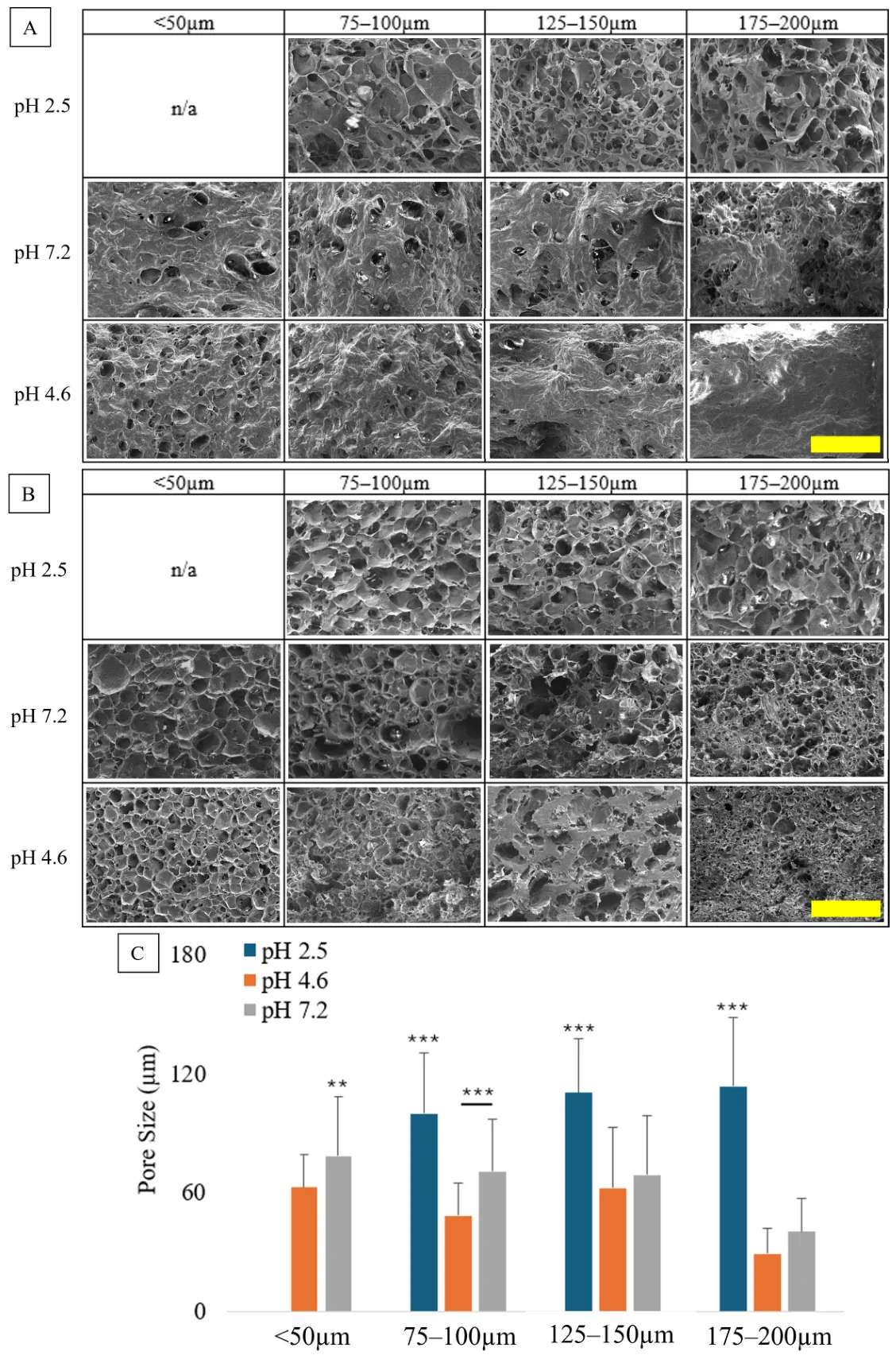
SEM and pore size analysis. (**A**) Surface and (**B**) cross-sectional views at 150× magnification of lyophilized, 2-layer crosshatch patterns of type I collagen microfiber-based inks 3D-printed using a 19G nozzle. For each microfiber size-range and hydration pH, formulations corresponding to achievable average densities after centrifuging at 14,000× *g* for 6 min were analyzed. Non-printable ink formulations are indicated by “n/a”. Scale bars: 300 µm. Low-magnification images are provided in Supplementary Materials Section S4, Figure S6. (**C**) Average cross-sectional pore size of the constructs (** *p* < 0.01; *** *p <* 0.001, *n* = 30–46, *t*-test).

Printed crosshatch patterns of type I collagen microfiber-based inks presented in Figure 5A were lyophilized and subsequently analyzed by SEM to further examine filament surface and cross-sectional morphology in greater detail (Figure 7). As shown in Figure 7A, surface pores were present that may provide cells and nutrients with access to the interior of the filaments, though the number generally decreased as the ink density increased. Interconnections between pores were also visible in the cross-sectional images (Figure 7B), which may further facilitate cell infiltration into the intrafilamentary space. Overall, the cross-sectional pore size decreased with increasing ink density as expected (Figure 7C).

In general, printing with the lowest-density ink formulations, obtained by hydrating microfibers at pH 2.5, resulted in significantly larger average pore sizes than with the higher-density ink formulations obtained by hydrating microfibers at pH 4.6 or 7.2. For the <50 µm and 75–100 µm microfiber size-ranges, printing with the highest-density ink formulation, achieved by hydration at pH 4.6, resulted in significantly smaller average pore sizes than with ink formulations at the other pH values. For the 125–150 µm and 175–200 µm microfiber size-ranges, the highest-density ink formulations obtained at pH 4.6 resulted in smallest average pore sizes, though no significant differences were observed compared to the ink formulations obtained at pH 7.2. Interestingly, low-magnification cross-sectional images (Supplementary Materials Section S4, Figure S6) show that stacked filaments form a continuous pore network rather than distinct boundaries between individual filaments. This may reduce the potential for printing-induced anisotropies in pore architecture that could influence direction-dependent cell behaviors, such as migration, compared to constructs whose filaments remain more distinct and separated.

#### 3.3.2. Effect of Collagen Microfiber Size and Density on Mechanical Properties

**Figure 8:**
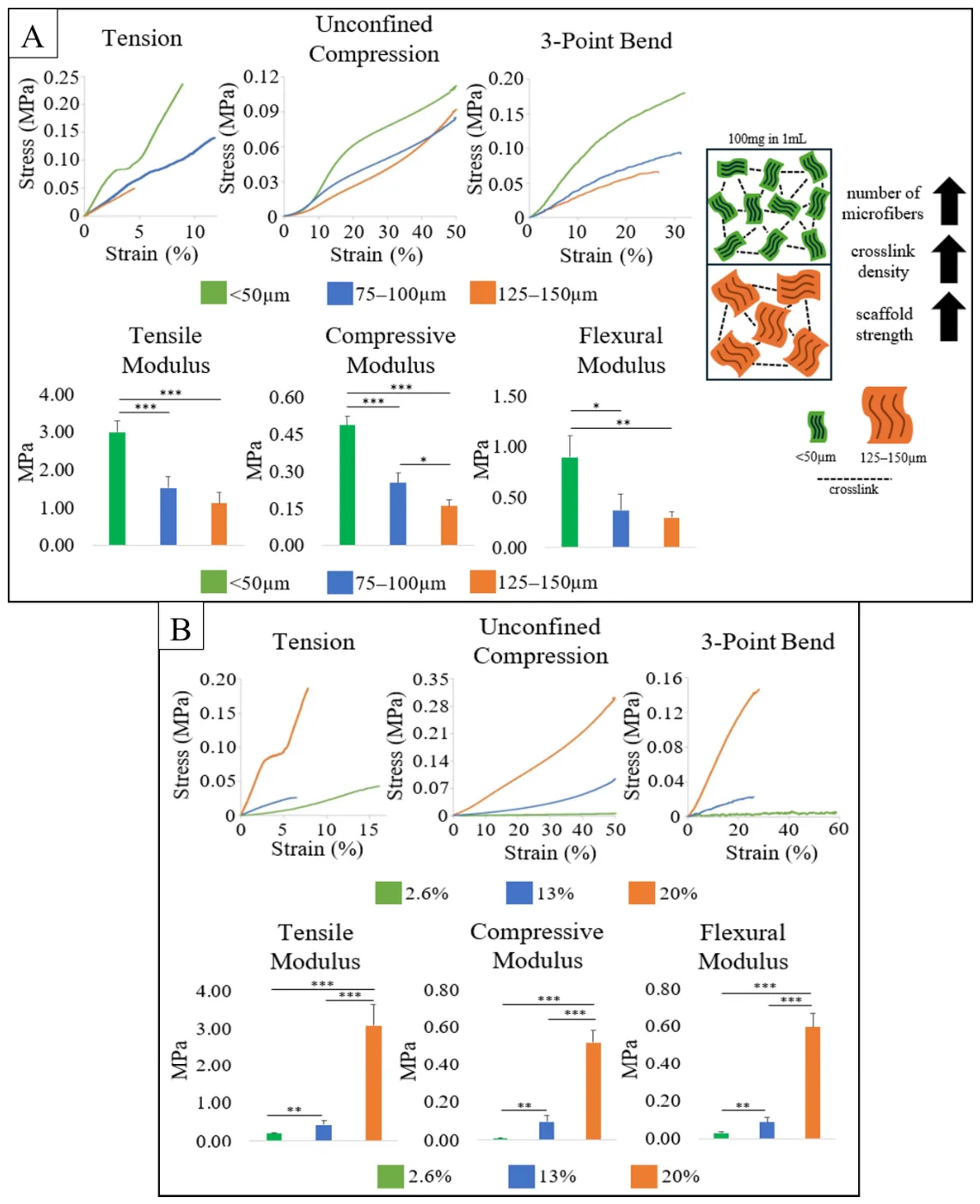
Effect of collagen microfiber size and density on scaffold mechanical properties. Average stress–strain curves (to ultimate stress) and moduli of scaffolds prepared from type I collagen microfiber-based inks with (**A**) a fixed collagen density (10%) and varying microfiber size-ranges and (**B**) a fixed microfiber size-range (175–200 µm) and varying collagen densities. Scaffolds were tested in tension, unconfined compression, and 3-point bending (* *p* < 0.05; ** *p* < 0.01; *** *p* < 0.001, *n* = 4, *t*-test).

For scaffolds (*n* = 4) fabricated at a fixed collagen density using different microfiber size-ranges (Figure 8A), the average modulus generally increased as the microfiber size-range decreased. Scaffolds fabricated from the smallest microfiber size-range exhibited a significantly higher average modulus across all tests. However, only in unconfined compression did scaffolds fabricated from the intermediate microfiber size-range exhibit a significantly larger average modulus than those fabricated from the largest microfiber size-range (*p* < 0.05). The average ultimate stress was also generally greatest for scaffolds fabricated from the smallest microfiber size-range; however, significant differences were observed only in tension and 3-point bending (*p* < 0.001) (Supplementary Materials Section S1.3, Figure S2A). Also, in unconfined compression, these scaffolds did not exhibit a significantly higher average ultimate stress compared to scaffolds fabricated from the largest microfiber size-range (*p* = 0.072). Scaffolds fabricated from the intermediate microfiber size-range generally exhibited higher average ultimate stress than those fabricated from the largest microfiber size-range in both tension and 3-point bending, but the difference was significant only in tension (*p* < 0.001). In contrast, during unconfined compression, scaffolds fabricated from the largest microfiber size-range exhibited a higher average ultimate stress than the intermediate microfiber size-range group, though this difference was not significant (*p* = 0.46) (Supplementary Materials Section S1.3, Figure S2A). Further analysis of the stress–strain curves showed that the average yield stress and toughness generally increased as the microfiber size-range decreased. Scaffolds fabricated from the smallest microfiber size-range exhibited significantly higher average yield stress and toughness than the other groups. However, scaffolds fabricated from the intermediate microfiber size-range exhibited significantly higher values than those fabricated from the largest microfiber size-range only in tension (Supplementary Materials Section S1.3, Figure S2A).

Even though the collagen content of the inks used to fabricate the scaffolds was similar, these results demonstrate differences in microfiber cohesiveness among different size-ranges within fabricated scaffolds after crosslinking. One possible explanation may be that the number of microfiber-to-microfiber crosslinks varies with microfiber size. Because individual smaller microfibers weigh less, a greater number of microfibers is required to achieve the same collagen weight compared to larger microfibers. Consequently, smaller microfiber size-ranges would be expected to form denser microfiber-to-microfiber networks after crosslinking, likely providing scaffolds’ greater resistances to deformation as illustrated in the schematic in Figure 8A.

Figure 8B shows the effect of collagen density on mechanical properties of scaffolds fabricated from the same microfiber size-range (*n* = 4). The high-density group exhibited higher average modulus across all tests (*p* < 0.001), while average moduli for the intermediate-density group were consistently significantly higher than average moduli for the low-density group (*p* < 0.01). The high-density group also exhibited the highest average ultimate stress in all tests (Supplementary Materials Section S1.3, Figure S2B). The intermediate-density group exhibited significantly higher average ultimate stress than the low-density group in unconfined compression and 3-point bending. In tension, the low-density group exhibited higher average ultimate stress than the intermediate-density group (*p* < 0.05). Further analysis of the stress–strain curves revealed that the low-density group was the most ductile, exhibiting an average failure strain roughly 3 times larger than the other groups in tension. Interestingly, during 3-point bending, all of the samples in the low-density group did not experience failure and the test was stopped when the sample began sliding down between supports. Although the low-density scaffolds were less stiff, their greater failure strain likely enabled them to sustain larger deformations, resulting in significantly higher ultimate stresses in tension compared to the intermediate-density group. The increased ductility of the low-density scaffolds may also explain why these samples did not fail during 3-point bending.

Average yield stress and toughness were generally significantly highest for the high-density group. However, in tension, toughness did not differ significantly between the high- and low-density groups (*p* = 0.22) (Supplementary Materials Section S1.3, Figure S2B). The intermediate-density group exhibited significantly higher average yield stress and toughness compared to the low-density group only in unconfined compression and 3-point bending. In tension, the greater ductility of the low-density scaffolds likely enabled them to exhibit significantly higher yield stress and toughness than the intermediate-density scaffolds. Overall, these results demonstrate that increasing collagen density generally enhances the mechanical strength of collagen-based scaffolds, while the increased ductility observed for the low-density group relative to higher-density groups may highlight a distinct mechanical behavior of intact, insoluble collagen microfibers hydrated at pH 2.5, which could provide a potential benefit for future scaffold design.

### 3.4. Cell Compatibility of High-Density Type I Collagen Scaffolds In Vitro

**Figure 9:**
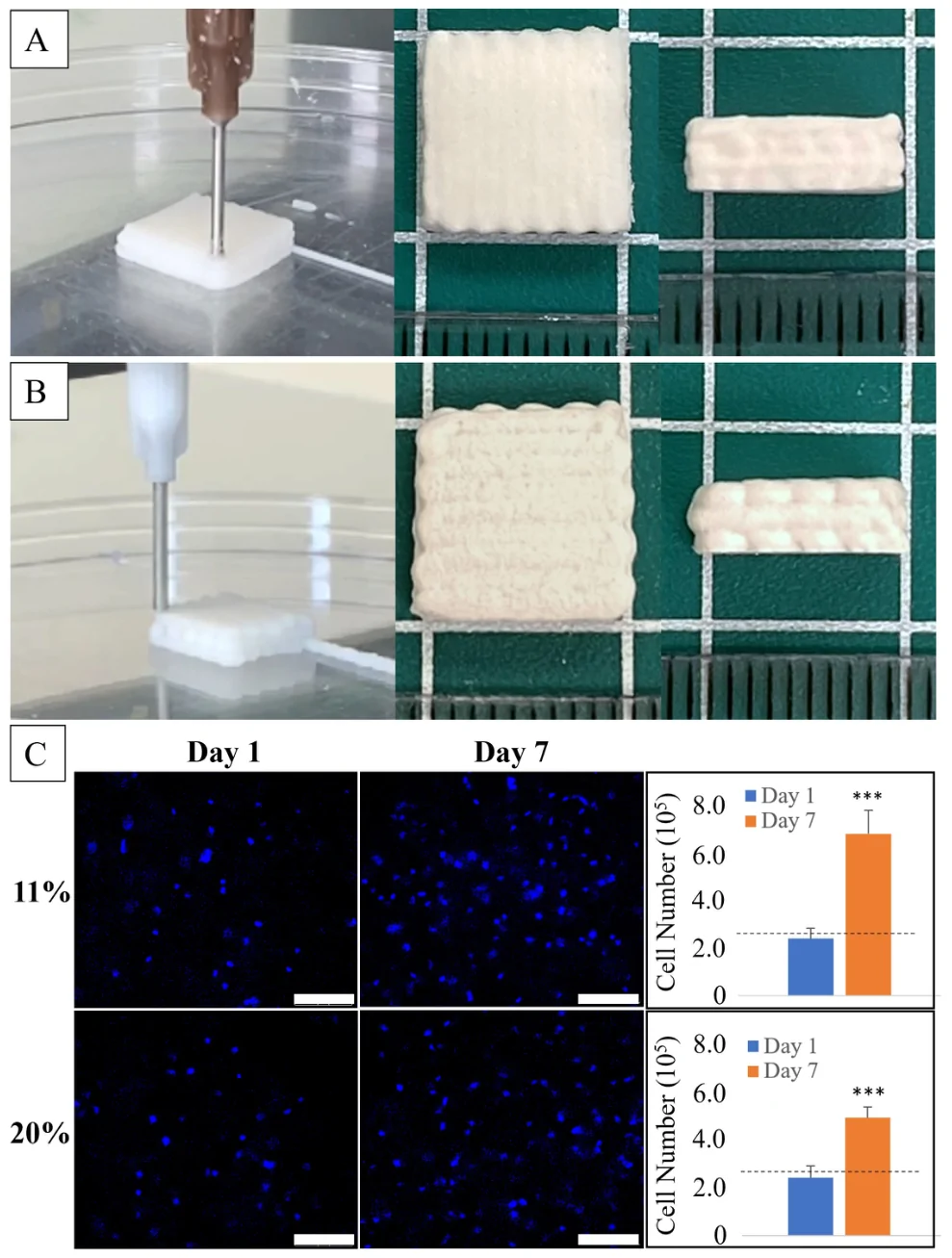
L929 fibroblast proliferation in scaffolds fabricated from high-density type I collagen microfiber-based inks. Solid blocks (*n* = 4) prepared from (**A**) 11% and (**B**) 20% density inks immediately after printing (**left**). Note the intentionally printed “tail” prior to the actual block being printed, which allows time for pressure within the syringe to stabilize following application of a force to the ink by the extrusion head. Top and side views of the printed blocks ((**middle**) and (**right**), respectively) after lyophilization shown with millimeter scale ruler. (**C**) Visualization of L929 fibroblasts by DAPI staining after 1 and 7 days in culture. Representative 20× images of stained cryosections (10 µm) ((**left**), Scale bars: 100 µm) and quantification of cell proliferation by Alamar Blue (**right**) following injection of L929 fibroblasts inside solid blocks prepared from 11% and 20% density inks. Dashed lines indicate the initial seeding amount (2.5 × 10^5^ cells) (*** *p* < 0.001, *n* = 4, *t*-test).

Figure 9 shows the proliferation of L929 fibroblasts in solid scaffolds fabricated from 11% and 20% density type I collagen microfiber-based inks. Proliferation was quantified using AlamarBlue and visualized by DAPI staining. Greater cell growth was observed in scaffolds prepared with the 11% density ink as expected (approximately 2.8-fold increase), since pore sizes are larger due to a lower collagen content compared to scaffolds prepared with the 20% density ink (Figure 7C). However, cells within the higher-density scaffold remained viable and still demonstrated an approximately two-fold increase in number over the culture period.

### 3.5. In Vitro Redifferentiation of Bovine Chondrocytes in High-Density Type II Collagen Scaffolds

**Figure 10:**
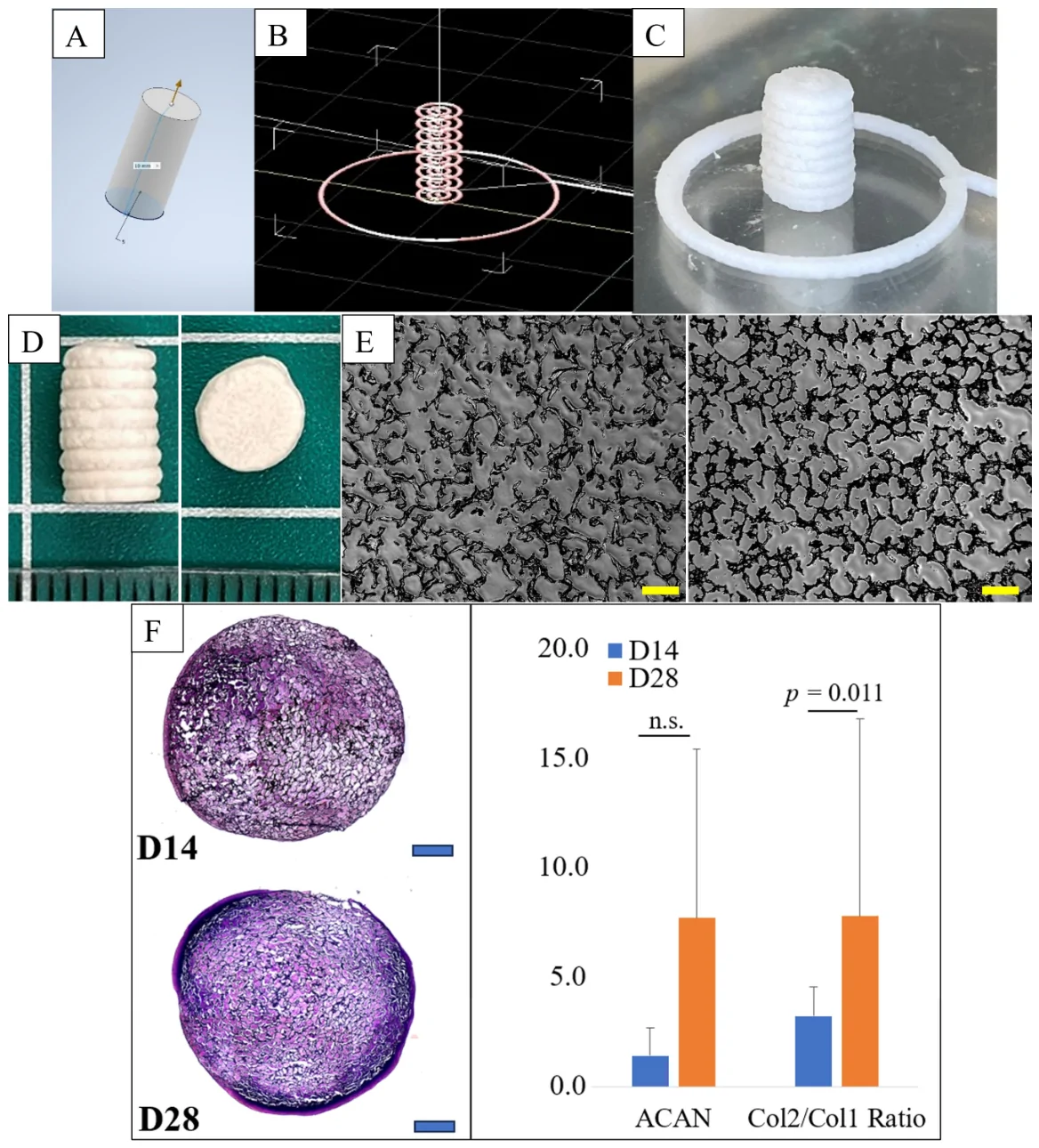
Chondrocyte redifferentiation in scaffolds fabricated from a high-density type II collagen microfiber-based ink. (**A**) Original cylinder designed in CAD software (AutoDesk^®^ Inventor^®^ Professional 2022 ver 26.0.15300.0000) exported as an STL file. (**B**) 3D visualization of the cylinder’s STL file after conversion to G-code using Repetrel (ver 4.2.540) slicing software. (**C**) Cylinder immediately after printing using a 15% density type II collagen microfiber-based ink. Note the intentionally printed “skirt” prior to the actual cylinder being printed, which allows time for pressure within the syringe to stabilize following application of a force to the ink by the extrusion head. (**D**) Side and top views of the 3D-printed cylinder after lyophilization shown with a millimeter-scale ruler. (**E**) Representative vertical cross-sections of 10 µm cryosections from 3D-printed (**left**) and manual injection-molded (**right**) cylinders at 10× magnification. Extrusion method did not affect pore structure. Pore sizes in samples used for the study ranged from approximately 50–150 µm, with an average ± standard deviation of 91 ± 59 µm. Scale bars: 100 µm. (**F**) P1 chondrocytes cultured in scaffolds (*n* = 6) fabricated from a 15% density type II collagen microfiber-based ink. Toluidine Blue staining of 10 µm cryosections for GAGs ((**left**), Scale bars: 1 mm) and relative gene expression of ACAN and the Col2/Col1 ratio after 14 and 28 days of culture in CDM ((**right**), *n* = 6, *t*-test; n.s.: not significant).

Dedifferentiated bovine chondrocytes (P1) cultured in scaffolds fabricated from a 15% type II collagen microfiber-based ink remained viable throughout the culture period (Figure 10F). Over the course of 28 days culture in CDM, GAG deposition increased throughout the scaffold cross section as indicated by the darker Toluidine Blue staining observed on day 28 compared with day 14. The P1 chondrocytes also exhibited signs of redifferentiation toward a hyaline chondrocyte-like phenotype, as demonstrated by increased average ACAN expression and a significant increase in the Col2/Col1 gene expression ratio. Overall, these results demonstrate the potential for high-density collagen scaffolds to support cell growth and function.

### 3.6. Potential Applications of High-Density Type I Collagen Inks

#### 3.6.1. Type I Collagen and Bone Mineral Composite Ink for Bone Regeneration

**Figure 11:**
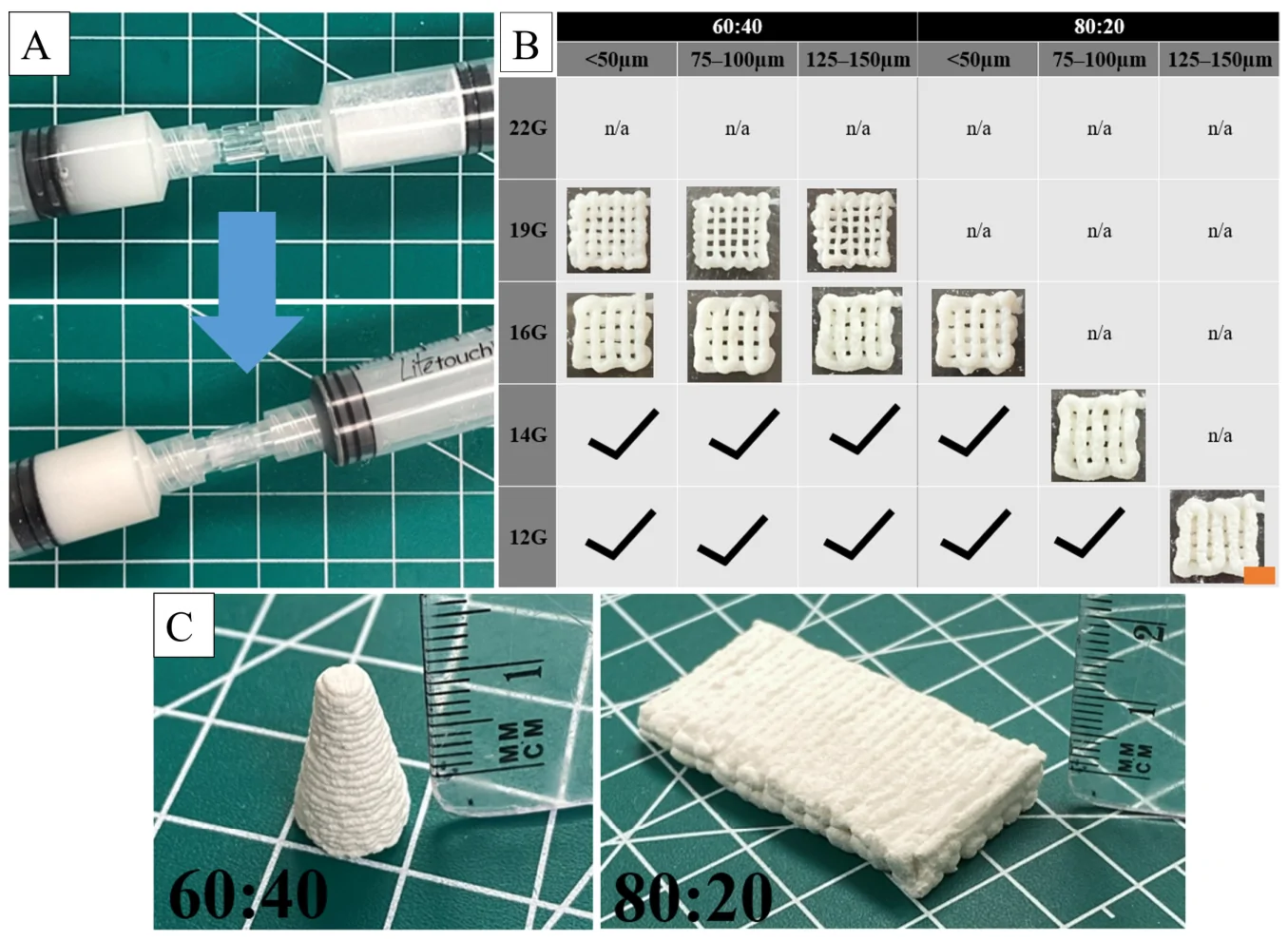
Preparation and printing of type I collagen microfibers plus bone mineral composite inks. (**A**) Incorporation of bone mineral into type I collagen microfiber-based inks. (**B**) Nozzle size suitability of type I collagen microfiber-bone mineral composite inks. Once the smallest printable nozzle size was determined, larger sizes were assumed to be printable as indicated by the checkmarks. Unsuccessful printing is indicated by “n/a”. (Scale bar: 5 mm). (**C**) Example scaffolds printed from type I collagen microfiber-bone mineral composite inks.

Preparation of a composite ink by mixing individual material components was demonstrated in Figure 11 with a high-density, type I collagen microfiber-based ink containing bone mineral. Preliminary printing results (Figure 11B) demonstrate nozzle sizes capable of extruding 60:40 and 80:20 (bone mineral:collagen) composite ink formulations. Once a minimum printable nozzle size was identified, it was assumed ink formulations were extrudable through subsequently larger nozzle sizes. Figure 11C presents example applications of printable bone mineral composite ink formulations. One example is a 60:40 composite ink printed into a conical plug (10 mm Ø × 15 mm height), which may be suitable for bone-grafting applications in dentistry. The second example is an 80:20 composite ink printed into a solid rectangular block (5 cm × 2 × cm 0.5 cm; L × W × H), which may serve as a scaffold for spine-fusion applications. Additional details regarding ink formulations and printing parameters are provided in Supplementary Materials Section S5.

#### 3.6.2. Type I Collagen Tube Fabrication

**Figure 12:**
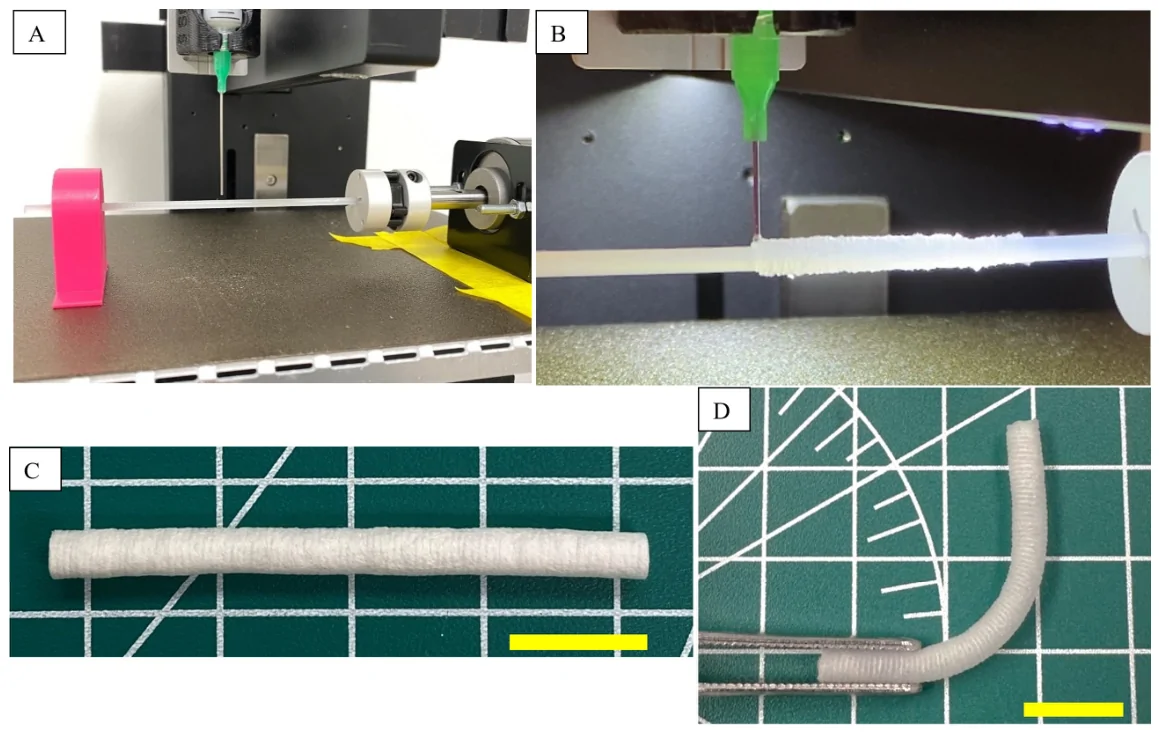
Fabrication of hollow tubes using a high-density type I collagen microfiber-based ink. (**A**) Extrusion setup in which a rod attached to a spinning motor was mounted on the print bed of the 3D printer. (**B**) Horizontal ink deposition onto the rotating rod to produce (**C**) a hollow tube after lyophilization. (**D**) Representative hydrated tube demonstrating bending flexibility after crosslinking (Scale bars: 1 cm).

Another potential application of high-density type I collagen microfiber-based inks was demonstrated in Figure 12. Figure 12A illustrates a setup for high-throughput fabrication of hollow tubes that leverages the extrudability of the inks. By directly extruding inks along a horizontally oriented rotating mandrel (Figure 12B), hollow tubes were obtained after lyophilization (Figure 12C). Because the tubes were formed from a single continuous filament, characteristic surface ridges (Figure 12C) were formed, which may contribute to mild kink-resistance during bending (Figure 12D). These high-density type I collagen tubes may be suitable for applications that require hollow, tubular structures such as nerve and vascular tissue engineering. Additional details regarding ink formulations and printing parameters are provided in Supplementary Materials Section S6.

## 4. Discussion

In the field of additive manufacturing, bioprinting is a newly developed technology, which enables 3D-printing of ECM scaffolds with simulated structures and compositions of native ECMs in vivo. Since collagen is the primary structural component of ECMs in most tissues, it is of the utmost importance to develop collagen-based inks for bioprinting applications with relevant collagen densities to the particular tissue of interest. However, the unique properties of collagen have precluded the development of such inks.

Reliance on monomeric, soluble collagen obtained from enzyme digestion has largely limited the application of type I collagen and type II collagen to hydrogel formulations typically containing no more than 1% (10 mg/mL) collagen [18,19,20]. This collagen content is insufficient for printing large, free-standing structures without extra supporting steps [26,27,28,29,30]. It has been shown that simply increasing the collagen in inks above 2% (20 mg/mL) improves printability, with recent advances in high-density collagen inks enabling soluble collagen densities up to 8% (80 mg/mL), while other groups have demonstrated the printing feasibility of non-enzyme-digested collagen including one group demonstrating 12.5% (125 mg/mL) [18,26,31,32,33,34,35]. However, these inks are still limited by either temperature control requirements, the need for stabilization steps, or collagen densities that remain lower than for weight-bearing tissues.

To address these limitations, we developed a collagen-based ink for extrusion-related applications, particularly bioprinting. By exploiting the inherent swelling property of intact, insoluble collagen fibers, inks were prepared at a wide range of densities featuring collagen that retained native fiber organization (since enzymatic digestion was not employed during the collagen purification process). To our knowledge, printing single-component collagen inks at 20% (200 mg/mL) as we have demonstrated at room temperature without extra stabilization steps represents the highest density reported for a single-component collagen ink so far. Collagen inks can also be prepared for a wide variety of tissue-engineering applications as successful extrusion and scaffold fabrication were demonstrated for both type I collagen and type II collagen. This capability eliminates the need to use type I collagen as a substitute when type II collagen is more appropriate [8,9].

For intact, insoluble collagen fibers to be extruded, they first must be able to pass through nozzles of various diameters. To achieve this, the macroscopic purified collagen fibers were reduced in size to microfibers through cryogenic grinding, which preserved native collagen structure as demonstrated through SDS-PAGE and thermal stability analysis (Figure 3). Even though DSC analysis shows the average thermal stability temperatures for the purified collagen materials were lower compared to their corresponding raw tissue (Figure 3C), the SDS-PAGE analysis did not show signs of damage to the collagen’s primary structure, and even the type I collagen microfibers still retained their native swelling ability. Decreases in thermal stability may likely have come from the removal of proteoglycans and non-collagenous proteins from raw tissues that otherwise would have contributed to the raw tissue’s overall thermal stability [46,47].

After hydrating the collagen microfibers, the amount of buffer removed after centrifuging will depend on how much of the buffer that microfibers retain under the hydration conditions, therefore determining the final collagen density of the inks. Even though we utilized 14,000× *g* to prepare inks, greater centrifugation forces could also be used if available (e.g., 20,000× *g*). It would be possible then, for example, that type I collagen microfibers less than 50 µm in size could be prepared at densities greater than 10%. If so, it is likely that every microfiber size-range and pH with which an ink is prepared would have a printable range of collagen densities. Further investigation to define these limits for each microfiber size-range would be necessary, but was beyond the scope of the present study. Instead, this work establishes a method to prepare high-density collagen-based inks composed of intact, insoluble collagen that can be extrusion-printed into 3D ECM scaffolds.

As mentioned above, ink preparation utilizes the electrostatic properties of collagen, particularly the effect of solution pH on the ionizable residues of Lysine, Arginine, Glutamic Acid, and Aspartic Acid [1,2,48,49,50]. At its isoelectric point (IP), the collagen molecule exhibits an equilibrium between positive and negatively charged residues within its triple helix, resulting in its most compact conformation as the charges attract each other. As the hydration buffer shifts away from the IP, this charge balance is disrupted, and the collagen molecule expands as electrostatic repulsion increases within the triple helix. In turn, collagen swells as the molecule can accommodate and retain more water than at its IP. Thus, the greater the charge imbalance, the greater the extent of molecular expansion and swelling of the collagen fibers. This principle was used to regulate the final collagen density by controlling microfiber swelling (Figure 4). To demonstrate the effect of pH on the swelling of the purified collagen material, we chose three pH conditions: pH 7.2, the IP (pH 4.6 for type I collagen and pH 5.0 for type II collagen), and pH 2.5. However, pH values other than the ones we chose in this study may also be used for ink optimization purposes.

At pH 7.2, cells can readily be incorporated with printed scaffolds either during or after printing. Therefore, it was important to demonstrate the straightforward preparation and extrusion of our collagen microfiber-based inks directly at this pH. In contrast, collagen hydrogels typically require a carefully controlled neutralization step to achieve cytocompatibility, or to form a physical structural network for cells to interact with [26,51]. Typically the IP of native type I collagen is approximately at a pH of 7.2 [49], indicating that the purification process (primarily the acid and base treatment steps) has some effect on the charged side chains and, therefore, on the IP. Our purified type I collagen from porcine tendon used in this study has an IP near pH 4.6 [unpublished data] and our purified type II collagen from bovine articular cartilage used in this study has an IP near pH 5.0 [unpublished data]. Therefore, these pH values were used to prepare the highest-density ink formulations. In acidic pH, it is well known that type I collagen will swell [50,52]. However, as we have demonstrated in the hydration of our intact, insoluble type II collagen microfibers (Figure 3A), the material does not swell to the same extent as type I collagen. To explain this behavior, we look towards well-established intermolecular and interfibrillar crosslinking patterns of native articular cartilage as further described below [53,54,55,56,57].

Type II collagen is the most abundant collagen in articular cartilage as it makes up the structural framework of the tissue along with minor amounts of type IX collagen and type XI collagen [53]. Though minor, type IX collagen and type XI collagen provide a critical contribution to the structural organization and mechanical stability of the type II collagen framework. Type XI collagen co-assembles with type II collagen, acting as a regulator of type II collagen fibril size, and type IX collagen has been hypothesized to provide covalent linkage sites between type II collagen fibrils and even proteoglycans [54,55]. Thus, the interaction of these three collagens within the articular cartilage ECM makes the collagen crosslinking architecture in the tissue rather complex. Due to the abundance of hydroxylysylpyridinolines (rather than lysyl oxidase-mediated crosslinks otherwise known as Schiff bases) primarily crosslinking the tissue [52,54,56,57], this complex collagen crosslinking architecture is also very stable. Pyridinolines are considered mature crosslink structures that actually arise from the condensation of Schiff bases, which are divalent, to form more stable trivalent linkages between collagen fibrils [52,54]. It is this abundance and stability of trivalent pyridinoline crosslinks stabilizing type IX collagen-mediated linkages throughout the collagen type II/type XI heterofibril network that enable articular cartilage to resist the swelling pressures from proteoglycans embedded in the ECM in vivo [52,54]. Since we have shown through our DSC and SDS-PAGE analysis that our type II collagen material purified from bovine articular cartilage retained native structure after purification and cryomilling (Figure 3 and [38]), preservation of the mature crosslinking network may explain why the type II collagen microfibers do not swell to the same extent as the type I collagen microfibers. This reduced swelling capacity likely contributed to the narrower range of obtainable densities for inks prepared from type II collagen microfibers compared to inks prepared from type I collagen microfibers (Figure 4).

We demonstrated that these microfiber-based inks generated well-defined filaments that remained intact during printing (Figure 5 and Supplementary Materials Section S3.1, Table S4) and maintained construct integrity, particularly at collagen densities greater than 2% (Figure 6B and Supplementary Materials Section S3.2, Figure S3E,F), without additional stabilization strategies such as post-printing NaCl precipitation of the collagen [35], UV crosslinking by incorporating light sensitive polymers into the ink (e.g., Gelatin Methacryloyl (GelMA) [27,28]), incorporating other additives such as alginate [29], or utilizing support hydrogels [26,30]. After post-printing processing (lyophilization and subsequent crosslinking), we demonstrated that controlling microfiber size and density provides a means to fine-tune physical and mechanical properties of the resulting scaffolds. For example, an ink prepared with a high density of microfibers < 50 µm in size produced scaffolds that were significantly stiffer after crosslinking than scaffolds fabricated with an ink at the same collagen density but of a larger microfiber size-range instead (Figure 8A). Optimization towards a specific tissue application would then involve identifying what composition of microfiber sizes in combination with crosslinking strategies would be most appropriate for the resulting scaffold to provide the desired mechanical properties. This finding highlights the potential for these microfiber-based inks to support diverse tissue repair and regeneration applications that require different scaffold mechanics [58].

To withstand their loading environments, native stiffnesses of weight-bearing tissues range from hundreds of kPa to tens of GPa [59,60]. Cartilage for example was reported as 0.41–0.85 MPa in compression and 5–25 MPa in tension. Reported values for bone range from 0.39 to 20.7 GPa [61]. For the meniscus, tensile modulus depending on the region of the tissue can reach up to 294 MPa [62]. Even though compressive and tensile moduli of scaffolds in this work did not fully reach certain native tissue stiffnesses (Figure 8), they nevertheless demonstrated improved mechanical properties compared to hydrogels also composed of solely collagen [19,31,32]. Scaffolds fabricated from high-density collagen microfiber-based inks may provide a more mechanically relevant starting point for developing scaffolds to treat injuries to weight-bearing tissues, and when combined with certain tissue-specific materials (e.g., hydroxyapatite for bone regeneration), their mechanical properties may be enhanced further [63,64].

When fabricating scaffolds, pore structure is an important design consideration because it is known to influence several key cellular processes such as migration, proliferation, and even differentiation [65]. Even though we demonstrated in Figure 7 the effect of ink density on pore size, relying solely on density for optimization may not be appropriate. Instead, controlling the freezing condition if the capability exists may provide a more effective strategy to tune pore size as well as morphology [66,67]. We chose to freeze directly at −20 °C prior to lyophilization with a “flask” style freeze-dryer to demonstrate a readily available workflow applicable to many laboratories. We recognize that further investigation is required to understand the design capabilities of these inks; however, such optimization should be reserved for specific scaffold development to a target application. The work presented here highlights the general feasibility of these high-density collagen microfiber-based inks as a scaffold fabrication material.

The 3D-printed ECM scaffolds serve as one of the three major components in tissue engineering research and tissue-equivalent implant development. The other two components are cells (tissue-specific cells or stem cells) and bioactive molecules (e.g., growth factors). For example, autologous chondrocyte implantation (ACI) has been used to treat articular cartilage injuries since the 1990s by injecting a cell suspension at the defect site which is then covered by a membrane [68,69]. A collagen-based scaffold has also been used in combination with autologous articular chondrocytes to improve repair outcomes (matrix-induced autologous chondrocyte implantation, MACI) [70,71]. However, MACI represents one example where type I collagen is used as a substitute in applications where type II collagen would be more physiologically relevant. The difficulty in utilizing intact, insoluble type II collagen to fabricate scaffolds stems from its limited swelling capacity as discussed earlier. Without swelling, the collagen reconstitution and engineering technologies common for intact, insoluble type I collagen are unable to be applied to intact, insoluble type II collagen, resulting in reliance on solubilization of native type II collagen in order to produce scaffolds [7]. In contrast, the microfiber ink preparation method described in this work bypasses the need to reconstitute fibers after swelling and homogenizing, enabling the fabrication of intact, insoluble type II collagen ECM scaffolds along with intact, insoluble type I collagen ECM scaffolds that better reflect the native collagen content of critical tissues in the human body [21,22,23,72].

When incorporating cells into scaffolds for tissue regeneration, either tissue-specific cells or stem cells can be directly seeded into prefabricated scaffolds via injection. Compared with relying on cell infiltration from surrounding healthy tissue, direct cell seeding into the interior of scaffolds can reduce the time required for cells to populate the scaffold. An alternative approach is to directly print tissue-equivalent constructs by incorporating cells into the ink [26,73,74,75,76,77]. Extruding cell-laden inks introduces additional considerations such as ink cytocompatibility, maintenance of a suitable microenvironment during and after printing, and sufficient structural integrity of printed constructs for handling and implantation. Therefore, inks must be near neutral pH to be compatible with the in vivo environment and support cell viability, and contain sufficient space to facilitate cell migration, proliferation, communication and nutrient transport.

For the collagen microfiber-based inks developed in this work, cells could theoretically be mixed directly with the collagen microfibers. However, the centrifugation required during ink preparation could compromise cell viability. Alternatively, ink formulations could be prepared separately and then mixed with a cell suspension prior to printing. However, this method would require careful adjustment of the collagen and cell concentrations to achieve the desired final ink formulation, and would not eliminate potential risks to cell viability as the cells could be exposed to mechanical stresses as the microfibers may deform, compress, and slide against one another during extrusion. A third approach would be to deposit cells separately from another nozzle directly onto printed filaments, which could help preserve cell viability by avoiding direct exposure to mechanical interactions with the microfibers during extrusion while maintaining the ink’s collagen density. Cell-laden extrusion printing represents a promising strategy for incorporating cells into tissue-equivalent constructs, and further investigation is required to assess the compatibility of our high-density, collagen microfiber-based inks with direct cell incorporation. In this study, we instead demonstrated cell incorporation into prefabricated 3D ECM scaffolds as this approach not only avoids exposing cells to the mechanical stresses associated with preparing and extruding highly concentrated collagen microfibers, but may also offer practical advantages for scaffold handling and implantation.

By following a general cell injection procedure, we have shown that scaffolds fabricated from high-density collagen microfiber-based inks support cell proliferation (Figure 9). This approach simplifies clinical translation and minimizes procedural complexity because implanting a solid, prefabricated scaffold requires minimal maneuvering compared to direct printing of a tissue-equivalent implant during surgery. Cells can then be incorporated into the prefabricated scaffold either prior to or after implantation via injection. This approach is particularly advantageous for load-bearing tissue applications such as in the knee. Instead of having to first print the tissue-equivalent implant, followed by possible further crosslinking and stabilization in situ which would add extra effort during surgery, scaffolds can be readily fabricated and mechanically reinforced and stabilized by chemical crosslinking in advance. Then during the actual surgery, prefabricated scaffolds would be immediately available “off-the-shelf” for implantation. Crosslinking prefabricated collagen scaffolds also provides an additional advantage.

Because collagen biomaterials are typically animal-derived, xenogeneic sources carry a potential risk of immune responses. The antigenic determinants capable of eliciting immune responses exist within both the triple-helical regions of collagen molecules and the non-helical telopeptide regions [78]. These determinants are more accessible within the telopeptides, providing rationale for their removal and the widespread use of atelocollagen in collagen-based tissue engineering strategies to reduce immunological responses. However, animal-derived collagen-based implants have consistently demonstrated high biocompatibility in humans even when using telopeptide-intact, insoluble collagen [1,78], suggesting that telopeptide removal provides a limited additional benefit [78]. Nevertheless, immunological risks remain as pre-existing hypersensitivity to bovine collagen is possible, as well as delayed allergy development even to atelocollagen [78]. In addition to telopeptide removal to reduce immunological responses, crosslinking has been shown to mask antigenic determinants [78,79,80,81]. This further increases the safety of animal-derived collagen-based implants, and provides additional support for using telopeptide-intact, insoluble collagen as a scaffold fabrication material especially when scaffolds are prefabricated and crosslinked prior to implantation as discussed above. In contrast, direct implantation of collagen scaffolds without crosslinking may compromise long-term in vivo performance. Without masking antigenic determinants and without crosslinking stabilization, risk of immune responses as well as implant failure increases since crosslinking is also used to tailor the rate of collagen degradation and resorption in the body [1,78,79,82]. Crosslinking therefore serves a dual role by improving biocompatibility and controlling scaffold stability, which are critical to the in vivo performance of collagen-based implants.

In addition to combining purified biomaterials to prepare a composite ink (e.g., collagen microfiber inks in combination with growth factors, cells, or tissue-specific components such as hydroxyapatite), the method described here can be adapted to prepare tissue-specific decellularized extracellular matrix (dECM) inks for bioprinting. Unlike collagen purification protocols that remove most non-collagenous proteins and other ECM components, dECM is prepared using milder processing methods that remove cell components such as cell debris, DNA, RNA, and other potential immunogenic materials (e.g., αgalactose-related epitopes) while preserving tissue-specific ECM components such as growth factors or glycosaminoglycans (GAGs). The resulting dECM would retain a composition that more closely resembles the native tissue at the microscopic level, therefore preserving tissue-specific biochemical and structural cues that may better support tissue-specific cell signaling [83,84,85,86,87,88]. Applying the ink preparation method described here to dECM could enable the development of dECM microfiber-based inks that retain advantages of the collagen microfiber-based inks discussed here, including the ability to directly extrude intact dECM at physiologically relevant collagen densities while preserving native ECM qualities without solubilization, or suspending solid dECM particles within hydrogel carriers to facilitate printing [86,87,88,89].

A limitation when utilizing dECM in this manner is the reduced ability to fabricate from the “bottom-up,” as the composition of printed scaffolds would reflect the overall composition of the source dECM from which the inks were prepared. Since dECM inks can be viewed as a naturally derived composite ink, the content of the dECM would also be highly dependent on the tissue processing. Furthermore, the native spatial organization of ECM components would be lost as microfibers are homogenously mixed during ink preparation. To address this, collagen microfiber-based inks can be used as a modular platform to first prepare tailored composite inks by incorporating selected components at defined concentrations. These composite inks can then be printed with other designed inks using multiple extrusion heads to fabricate scaffolds with spatially controlled compositions.

Whether prepared from tissue-specific dECM, from tailored composite formulations, or from collagen microfibers alone, inks prepared using the method described in this study have the potential to support various tissue engineering applications. As demonstrated in Figure 10, type II collagen scaffolds fabricated from high-density type II collagen microfiber-based inks can be utilized for cartilage tissue engineering. Figure 11 and Figure 12 illustrate additional potential applications, including scaffolds for bone regeneration fabricated from type I collagen microfiber-based inks containing calcium carbonate mineral particles, and hollow type I collagen tubes that could serve as nerve guidance conduits for peripheral nerve regeneration or vascular grafts for blood vessel repair.

## 5. Conclusions

In summary, our high-density collagen microfiber-based biomaterial inks offer several unique advantages, including preserved native collagen fiber architecture, collagen densities that better reflect those of various type I- and type II collagen-rich tissues, direct preparation at neutral pH to produce physiologically relevant inks or render prefabricated scaffolds cell-compatible, and straightforward incorporation of single or multiple components with collagen microfibers to generate composite inks for “ground-up” fabrication of ECM scaffolds via multi-nozzle extrusion printers. These tunable inks hold promise for 3D extrusion printing of scaffolds for various tissue engineering applications including cartilage, bone, nerve, or vascular regeneration.

## Data Availability

The original contributions presented in this study are included in the article/Supplementary Materials. Data supporting findings of this study are available from the corresponding author upon reasonable request.

## Supplementary Materials

The following supporting information can be downloaded at: https://www.mdpi.com/article/10.3390/polym18182261/s1, Table S1: Quality analysis of purified insoluble type I collagen fibers; Figure S1: Hydrated solid block scaffolds; Table S2: Scaffold preparation parameters for mechanical testing; Figure S2: Effect of collagen microfiber size and density on mechanical properties; Table S3: TaqMan Primers and Assay ID; Table S4: Quantitative printability metrics; Figure S3: Rheological evaluation of collagen microfiber-based inks; Figure S4: Printed 4-layer crosshatch patterns; Figure S5: Pore closure analysis; Figure S6: Low-magnification SEM images. Refs. [38,45] are cited in the Supplementary File.
