# Supplementary material for: High-Density, Extrudable Collagen Types I and II Microfiber-Based Inks for Tissue Engineering Applications
Source: Polymers (Basel). 2026 Sep 16;18(18):2261. doi: 10.3390/polym18182261 (PMC13611240; doi:10.3390/polym18182261)
Supplement: Supplementary file 1 [file polymers-18-02261-s001.zip › polymers-4418335-supplementary.pdf]

Polymers-4418335R1

Supplementary Materials

HIGH-DENSITY, EXTRUDABLE COLLAGEN TYPES I AND II MICROFIBER-BASED INKS FOR TISSUE ENGINEERING APPLICATIONS

Kevin Capariño, MSE[1] and Shu-Tung Li, PhD[1]*
Shu-Tung and Alice Li Foundation, Laboratory for Tissue Engineering Research[1]
Oakland, NJ 07436, USA
*Correspondence: sli@thelifoundation.org

1. 3D-Printed High-Density Type I Collagen Microfiber-Based Scaffolds

1.1 Quality Analysis of Insoluble Type I Collagen fibers

To assess the purity and batch-to-batch variability of the purified insoluble, type I collagen fibers, we performed Differential Scanning Calorimetry (DSC) analysis and a series of chemical assays—hydroxyproline content, DNA quantification, and uronic acid content—as previously described [1]. Our DSC and chemical analyses of 4 different batches of insoluble type I collagen fibers (Supplementary Table S1) demonstrated high purity and minimal batch-to-batch variability in the insoluble collagen fibers isolated from porcine tendon.

Table S1: Quality analysis of purified insoluble type I collagen fibers.

Quality Analysis	Lot 1 (n=3)	Lot 2 (n=3)	Lot 3 (n=3)	Lot 4 (n=3)	Mean ± SD
Hydroxyproline (%)	13.96 ± 0.15	13.67 ± 0.34	13.67 ± 0.25	13.76 ± 0.39	13.77 ± 0.14
DNA (ng/mg)	ND	ND	ND	ND	ND
Uronic Acid (%)	0.09 ± 0.02	0.03 ± 0.05	0.07 ± 0.02	0.05 ± 0.02	0.06 ± 0.03
Denaturation temperature (Td, °C)	61.2 ± 1.4	61.6 ± 1.2	62.1 ± 1.5	60.5 ± 0.3	61.4 ± 0.7

ND: non-detectable

1.2 Type I Collagen Microfiber-Based Scaffolds for Mechanical Testing

Type I collagen solid block scaffolds were printed with a 19G blunt-tipped nozzle by depositing filaments in a crosshatch pattern with no gaps between adjacent filaments. Ink formulations included a fixed collagen density of 10% with varying microfiber size-ranges, and a fixed microfiber size-range of 175-200µm with different collagen densities (approximately 2.6%, 13%, and 20%). Supplementary Figure S1 shows representative hydrated scaffolds used for tensile, three-point bending, and unconfined compression testing as shown in Figure 2. Supplementary Table S2 summarizes the ink formulations and printing parameters.

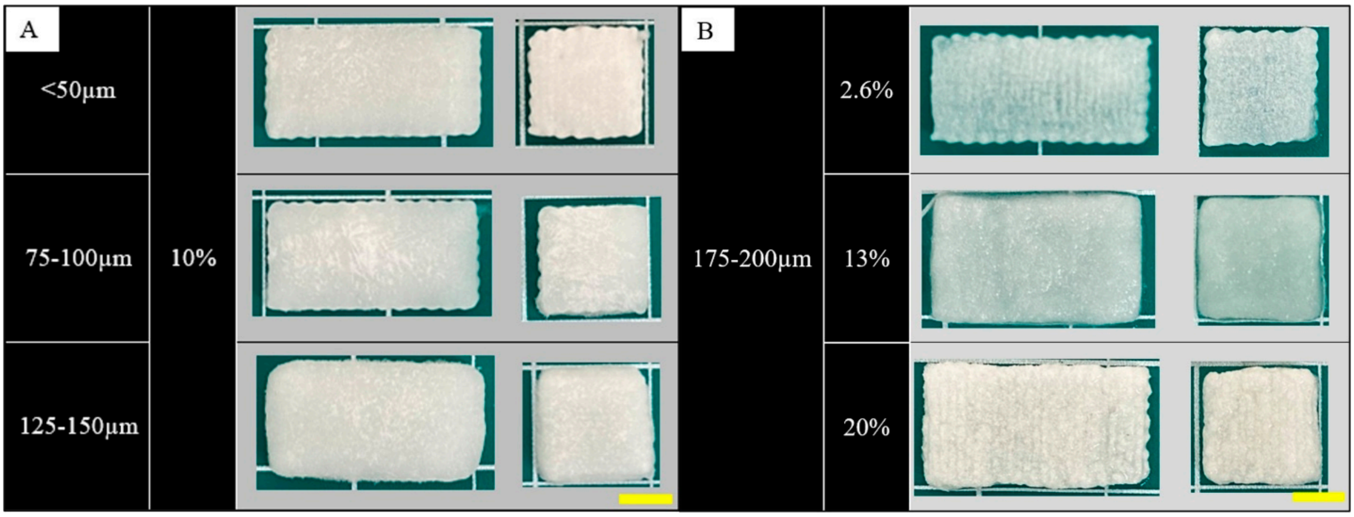

Figure S1: Hydrated solid block scaffolds. Scaffolds fabricated with (A) 10% collagen density inks containing different microfiber size-ranges and (B) inks consisting of varying densities of 175-200µm type I collagen microfibers. Scale bars: 5mm.

Table S2: Scaffold preparation parameters for mechanical testing. Hydration pH, centrifugation parameters, printing speed (PS), and calculated extrusion flow rate (FR).

Microfiber Size	Hydration pH	Approximate Collagen Content	Centrifuge Parameters	Printing Parameters
<50µm	4.6	10%	14k x g ; 6mins	PS: 800-860 mm/min<br>FR: 9.6-10.4 µl/sec
75-100µm			8k x g ; 1min	PS: 900-960 mm/min<br>FR: 10.8-11.6 µl/sec
125-150µm			500 x g ; 1min	PS: 4000-4600 mm/min<br>FR: 48.2-55.4 µl/sec
175-200µm	2.5	2.6%	14k x g ; 6mins	PS: 1000-1400 mm/min<br>FR: 12.0-16.9 µl/sec
	4.6	13%	1k x g ; 1min	PS: 4500-5000 mm/min<br>FR: 54.2-60.2 µl/sec
		20%	14k x g ; 6mins	PS: 2000-2400 mm/min<br>FR: 23.0-27.6 µl/sec

1.3 Ultimate Stress, Yield Stress, and Toughness

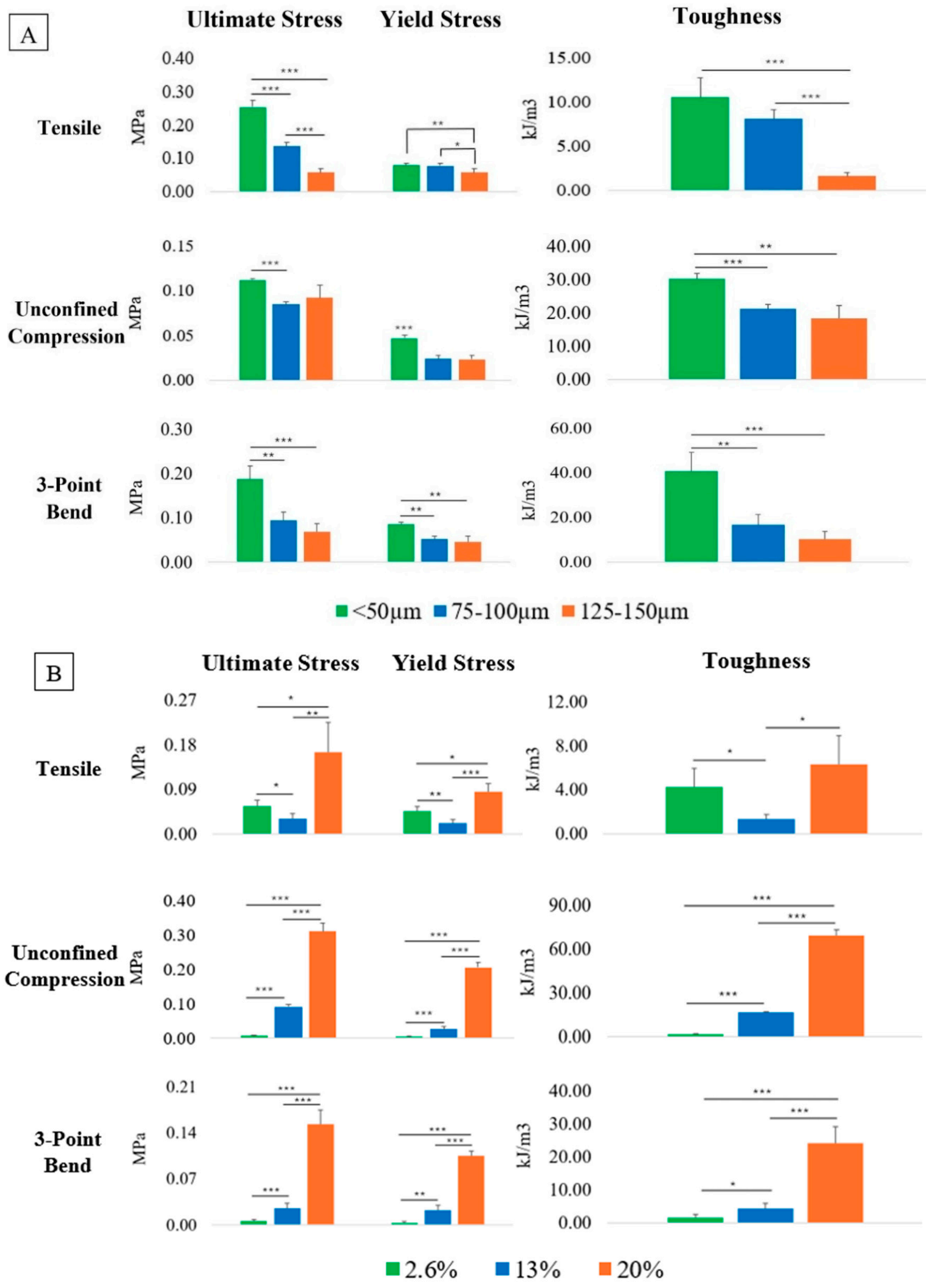

Figure S2: Effect of collagen microfiber size and density on mechanical properties. Average ultimate stress, yield stress, and toughness of (A) type I collagen microfiber-based scaffolds at a

fixed collagen density (10%) with varying microfiber size-ranges (<50µm, 75-100µm, and 125-150µm) and (B) scaffolds at a fixed microfiber size-range (175-200µm) with varying collagen densities tested in tension, unconfined compression, and 3-point bending (**p*<0.05; ** *p* <0.01; *** *p* <0.001, n=4, t-test).

The effect of microfiber size and collagen density on Ultimate Stress, Yield Stress, and Toughness are shown in Supplementary Figure S2. Ultimate Stress was determined from stress-strain curves by identifying the maximum stress value, which corresponded to failure stress in tensile and 3-point bending testing. Yield Stress was determined from stress-strain curves using the 0.2% offset method to identify the stress at which samples began to undergo plastic deformation. Toughness was approximated from stress-strain curves using the Trapezoidal Rule integration method in 2.5%-5% strain increments, depending on the shape of the curve, to quantify the energy absorbed before failure or up to the highest strain reached. The average Ultimate Stress, Yield Stress, and Toughness of scaffolds fabricated with <50µm type I collagen microfibers were significantly higher than those of the larger microfiber groups (75–100µm and 125–150µm). In scaffolds composed of 175–200 µm microfibers, the average Ultimate Stress, Yield Stress, and Toughness of scaffolds generally increased with collagen density.

1.4 Type I Collagen Microfiber-Based Scaffolds for Cell Culture

Type I collagen solid block scaffolds (L x W x H; 1cm x 1cm x 0.4cm) were printed using 11% and 20% type I collagen microfiber-based inks deposited in a crosshatch pattern with no gaps between filaments. The 11% inks were prepared using 75-100µm microfibers, hydrated in PBS at a concentration of 1% (w/v) for 3hrs, and centrifuged at 14,000 × g for 6 minutes. This ink was extruded using a 19G blunt-tipped nozzle at a printing speed of 1600-1800 mm/min, corresponding to a calculated extrusion flow rate of approximately 17.5-19.6 µl/sec. The 20% inks were prepared using 175-200 µm microfibers, hydrated in 0.1M sodium acetate buffer (pH 4.6) at a concentration of 1%(w/v) for 3hrs, and centrifuged at 14,000 × g for 6 minutes. This ink was extruded using an 18G blunt-tipped nozzle at a printing speed of 600 mm/min, corresponding to a calculated extrusion flow rate of approximately 14.4 µl/sec. After lyophilization, the solid block scaffolds were crosslinked for cell culture as described in Section 2.5 of the main text*: Post-Printing Processing and Scaffold Characterization.*

2. 3D-Printed High-Density Type II Collagen Microfiber-Based Scaffolds

2.1 Type II Collagen Scaffolds for Cell Culture

A 15% density type II collagen microfiber-based ink was prepared using microfibers 25-50µm in size, hydrated in 0.1M sodium acetate buffer (pH 5.0) at a concentration of 1% (w/v) for 1hr and centrifuged at 14,000×g for 6 minutes. The ink was extruded using a 17G blunt-tipped nozzle at a printing speed of 500 mm/min, corresponding to a calculated extrusion flow rate of approximately

10.1 µl/sec. After lyophilization, the cylinders were crosslinked for cell culture as described in Section 2.5 of the main text*: Post-Printing Processing and Scaffold Characterization*. A second cylinder was prepared by manual injection-molding the same ink into a 96-well plate, followed by freeze-drying and crosslinking under identical conditions, to assess whether the extrusion process influenced pore structure as shown in Figure 10 of the main text.

Pore structure was evaluated histologically by fixing crosslinked cylinders in 10% neutral buffered formalin (Sigma-Aldrich) overnight, followed by sequentially dehydrating in 15% sucrose (Fisher Scientific) in Phosphate Buffered Saline (PBS) (Gibco) until samples sank, and then 30% sucrose in PBS until samples sank. After dehydration, samples were embedded in Tissue-Plus O.C.T. Compound (Fisher Scientific) for at least 12hrs prior to sectioning. Cryosections (10µm) were prepared using a Cryostat Microtome (Thermo Scientific HM550), mounted on Fisherbrand Superfrost Plus Microscope Slides (Fisher Scientific), and then imaged using a VWR microscope equipped with a digital color camera. Image analysis showed similar pore morphology between the 3D-printed cylinder and the manual injection-molded cylinder (Figure 10E). Pore sizes of samples used in this study generally ranged from 50 to150µm (average 91 ± 59 µm).

2.2 Primary Bovine Chondrocyte Isolation and Culture

Primary bovine chondrocytes were isolated from the articular cartilage of the femoral heads of 4.5-month-old calves (Spear Products Inc., Coopersburg, PA). Shaved cartilage slices were minced into ~1 mm^3 pieces and digested overnight by 0.25% collagenase II (310 U/mg; Gibco, Thermo Fisher Scientific) in high-glucose DMEM (Gibco) supplemented with 10% FBS and 1% penicillin–streptomycin at 37 °C and 5% CO_2 under gentle agitation.

After digestion, the suspension was filtered with a 70µm Falcon cell strainer (Corning Life Sciences, Tewksbury, MA) to remove undigested tissue. After centrifuging at 1500rpm to collect the cells, primary bovine chondrocytes were seeded at a density of approximately 1×10^5 cells/cm^2 and cultured in growth medium at 37°C in a humidified atmosphere with 5% CO_2 until reaching 80–90% confluence, which was defined as passage 0 (P0) bovine chondrocytes. After reaching confluence, the P0 bovine chondrocytes were expanded via trypsinization and subculturing by seeding at 9×10^3 cells/cm^2. After reaching 80–90% confluence in T-175 flasks (Fisherbrand, Fisher Scientific), the dedifferentiated bovine chondrocytes (P1) were trypsinized and injected into scaffolds fabricated with a 15% density type II collagen microfiber-based ink at a density of 1×10^6 cells/scaffold using a 29G needle syringe.

After 7 days in growth medium under a normoxic (21% O_2) and humidified atmosphere containing 5% CO_2, the cell-laden scaffolds were cultured in chondrocyte differentiation medium (CDM) under dynamic conditions (100RPM orbital rotation) in a hypoxic incubator (5% O_2). CDM consisted of high-glucose DMEM (Gibco) supplemented with 1% ITS+1 liquid media supplement (Sigma-Aldrich), 100 µg/mL sodium pyruvate (Sigma-Aldrich), 50 µg/mL ascorbic acid-2-phosphate (Sigma-Aldrich), 40 µg/mL L-proline (Sigma-Aldrich), 0.1µM dexamethasone (Sigma-Aldrich), and 10 ng/mL TGF-β3 (PeproTech, Thermo Fisher Scientific). Scaffolds then were harvested after 2 and 4 weeks in CDM for histological and qRT-PCR analyses.

2.3 Toluidine Blue Staining for Glycosaminoglycans (GAGs)

At each experimental timepoint, cell-laden collagen scaffolds were fixed in 10% neutral buffered formalin (Sigma-Aldrich) for at least 24 hours at 4°C. Fixed scaffolds were then sequentially incubated at 4°C in 15% sucrose (Fisher Scientific) in Phosphate Buffered Saline (PBS) (Gibco) until sinking, followed by 30% sucrose in PBS until sinking. Finally, the scaffolds were embedded in Tissue-Plus O.C.T. Compound (Fisher Scientific). 10 µm thick sections from the embedded scaffolds were then sectioned using a Cryostat Microtome (Thermo Scientific HM550) and mounted on Fisherbrand Superfrost Plus Microscope Slides (Fisher Scientific). After air-drying, the sections were stained for 2 min with 0.5% toluidine blue-O (pH 4.0) (Sigma Aldrich). After washing the stained slides with tap water and air-drying, slides were examined using a VWR microscope equipped with a digital color camera.

2.4 RNA Isolation and qRT-PCR

At each experimental timepoint, cell-laden scaffolds were removed from CDM, blotted semi-dry with lint-free Kimwipes, and transferred into autoclaved 1.5mL microcentrifuge tubes with 600 µL of lysis buffer per scaffold (GeneJET RNA Purification Kit, Thermo Fisher Scientific). Scaffolds were then homogenized with a pestle motor homogenizer (Cole-Parmer) until a uniform suspension was obtained. The suspension was incubated at room temperature for at least 10 minutes and then frozen at –80°C overnight.

The next day, samples were thawed at room temperature and vortexed. The lysates were then centrifuged at 12,000 rpm at 4°C (Beckman Coulter, Brea, CA) to remove scaffold debris, and the supernatants (chondrocyte lysates) were transferred to new autoclaved 1.5mL microcentrifuge tubes. RNA was isolated from the supernatant with the GeneJET RNA Purification Kit following manufacturer's instructions. Total RNA yield was quantified by measuring absorbance at 260 nm and 280 nm using a microplate reader (SpectraMax M2, Molecular Devices).

Real-time quantitative reverse transcription PCR (qRT-PCR) was performed with an AriaMx Real-Time PCR Instrument (Agilent Technologies, Santa Clara, CA) using predesigned TaqMan FAM-MGB probes and primers of target genes (Thermo Fisher Scientific), as shown in Supplementary Table S3, and TaqPath 1-Step Multiplex Master Mix (Applied Biosystems,Thermo Fisher Scientific). The relative expression of each target gene was normalized to *GAPDH* as the housekeeping gene using the ΔCt method, where $\Delta Ct = Ct_{gene} - Ct_{GAPDH}$. Normalized values of $2^{-\Delta Ct}$ were compared between groups.

Table S3: TaqMan Primers and Assay ID.

Gene symbol	TaqMan Assay ID
Col1a1	Bt01463861_g1
Col2a1	Bt03251861_m1
ACAN	Bt03212186_m1
GAPDH	Bt03210913_g1

3. *Quantitative Printability Metrics and Construct Integrity*

3.1 Filament Spreading Ratio and Pore Fidelity Determination

Filament spreading ratio was defined as the ratio of the measured filament width to the nozzle diameter used (n=10 filaments per sample), where values closer to 1 indicate greater shape fidelity. Pore closure and shape fidelity analysis (n=10 pores)[2] were performed using ImageJ software. Filament spreading ratios (FS) and pore shape fidelity values (PF) of type I collagen and type II collagen microfiber-based inks, printed into crosshatch patterns for 2 layers as demonstrated in Figure 5, were shown below in Supplementary Table S4.

Table S4: Quantitative printability metrics.

Type I Collagen

pH 4.6	<50μm (9.8%)	75-100μm (12.9%)	125-150μm (15.3%)	175-200μm (20.2%)
22G	FS: 1.05 ± 0.17<br>PF: 0.94 ± 0.08	n/a	n/a	n/a
19G	FS: 1.01 ± 0.10<br>PF: 0.86 ± 0.06	FS: 0.97 ± 0.09<br>PF: 0.92 ± 0.06	FS: 1.07 ± 0.11<br>PF: 0.94 ± 0.16	FS: 0.99 ± 0.15<br>PF: 0.99 ± 0.10
16G	FS: 0.92 ± 0.10<br>PF: 0.97 ± 0.1	FS: 1.03 ± 0.09<br>PF: 0.84 ± 0.09	FS: 0.89 ± 0.08<br>PF: 0.95 ± 0.07	FS: 0.93 ± 0.12<br>PF: 0.94 ± 0.10
**pH 7.2**	**<50μm (8.9%)**	**75-100μm (11.4%)**	**125-150μm (12.8%)**	**175-200μm (16.8%)**
22G	FS: 1.11 ± 0.17<br>PF: 0.84 ± 0.04	FS: 1.18 ± 0.15<br>PF: 0.84 ± 0.05	n/a	n/a
19G	FS: 1.14 ± 0.14<br>PF: 0.86 ± 0.04	FS: 1.04 ± 0.14<br>PF: 0.84 ± 0.03	FS: 0.93 ± 0.11<br>PF: 0.89 ± 0.04	FS: 1.10 ± 0.21<br>PF: 1.01 ± 0.13
16G	FS: 0.92 ± 0.07<br>PF: 0.94 ± 0.14	FS: 1.10 ± 0.08<br>PF: 0.87 ± 0.09	FS: 0.99 ± 0.09<br>PF: 0.87 ± 0.08	FS: 1.01 ± 0.06<br>PF: 0.97 ± 0.22
**pH 2.5**	**<50μm (n/a)**	**75-100μm (2.3%)**	**125-150μm (2.2%)**	**175-200μm (2.6%)**
22G	n/a	FS: 1.12 ± 0.14<br>PF: 0.91 ± 0.13	FS: 1.14 ± 0.22<br>PF: 1.01 ± 0.12	FS: 1.04 ± 0.23<br>PF: 1.05 ± 0.21
19G	n/a	FS: 0.93 ± 0.09<br>PF: 1.15 ± 0.21	FS: 1.01 ± 0.16<br>PF: 0.96 ± 0.06	FS: 0.88 ± 0.13<br>PF: 1.20 ± 0.23
16G	n/a	FS: 0.88 ± 0.09<br>PF: 1.04 ± 0.15	FS: 1.00 ± 0.14<br>PF: 0.93 ± 0.56	FS: 0.96 ± 0.09<br>PF: 1.06 ± 0.14

Type II Collagen

pH 5.0	<50μm (14.3%)	75-100μm (15.5%)	125-150μm (17.2%)	175-200μm (20.3%)
22G	n/a	n/a	n/a	n/a
19G	FS: 1.08 ± 0.12<br>PF: 0.84 ± 0.04	FS: 1.08 ± 0.14<br>PF: 0.91 ± 0.09	FS: 1.06 ± 0.14<br>PF: 0.99 ± 0.15	FS: 1.10 ± 0.14<br>PF: 0.91 ± 0.21
**pH 7.2**	**<50μm (11.9%)**	**75-100μm (13.3%)**	**125-150μm (15.0%)**	**175-200μm (18.1%)**
22G	FS: 1.18 ± 0.14<br>PF: 0.81 ± 0.06	FS: 1.13 ± 0.14<br>PF: 0.87 ± 0.05	n/a	n/a
19G	FS: 1.12 ± 0.11<br>PF: 0.80 ± 0.05	FS: 1.07 ± 0.11<br>PF: 0.84 ± 0.06	FS: 1.07 ± 0.12<br>PF: 0.90 ± 0.09	FS: 1.13 ± 0.12<br>PF: 1.01 ± 0.22

3.2 Rheology

Rheological testing was performed by Fluid Dynamics Testing LLC (Flemington, NJ). Frequency Sweep (0.01-100 radians/s ; 5 points per decade) and Strain Sweep (0.001-1 ; 10Hz ; 10 points per

decade) testing were performed in the 25 mm parallel plate geometry with 0.8-1.0 mm spacing. Spacing increased to 1.0-1.34 mm to accommodate higher-density samples as needed. Samples were kept in a "closed" trap during testing with humidified padding on the end caps. Tests were performed at 20°C after 60 sec thermal equilibration from sample loading to the start of the test.

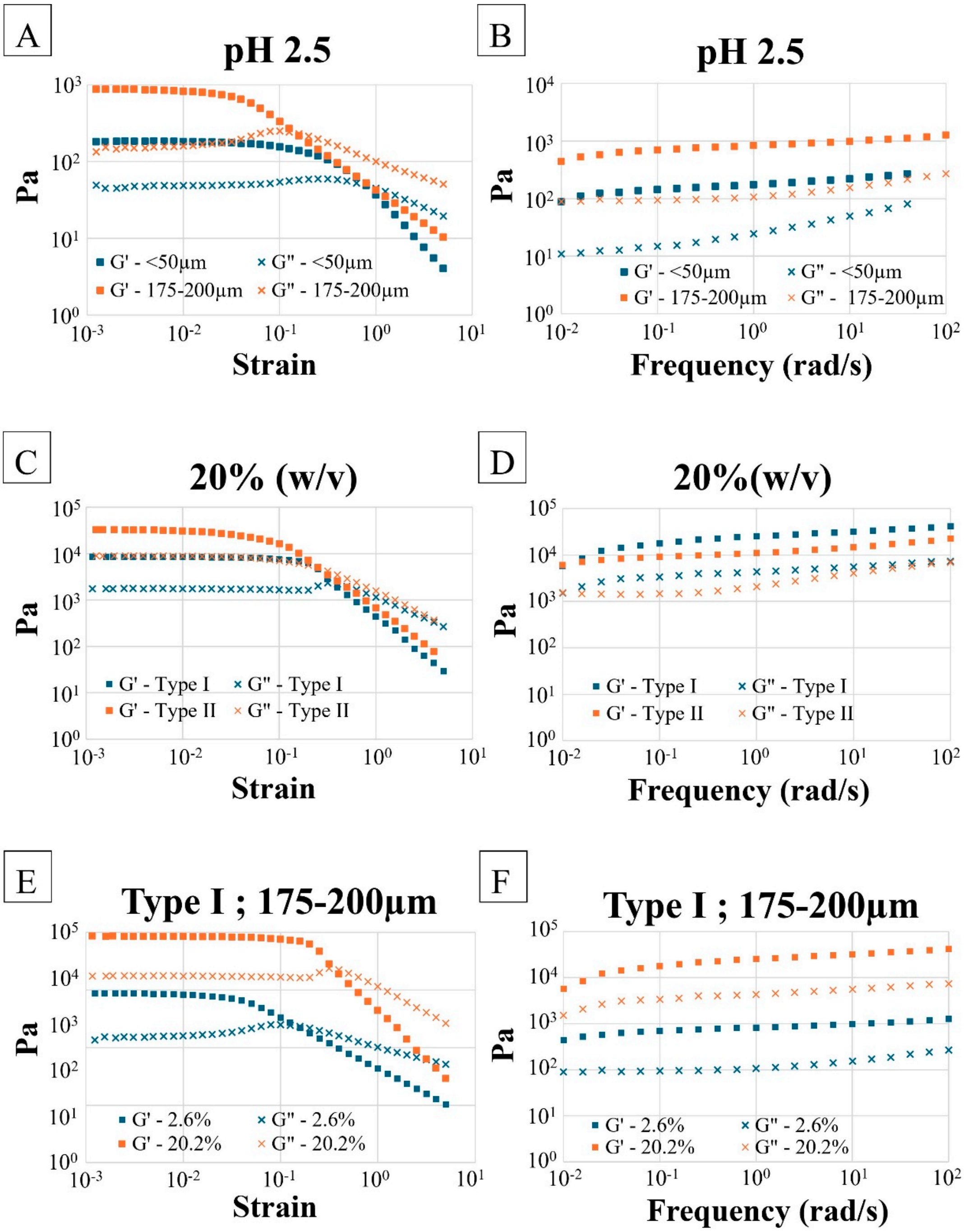

Figure S3: Rheological evaluation of collagen microfiber-based inks. Effect of (A,B) microfiber size, (C,D) collagen type, and (E,F) collagen density on rheological properties of collagen microfiber-based inks. (A,C,E) Strain and (B,D,F) frequency sweeps performed at 20°C.

The rheological properties of the non-printable ink formulation, <50 μm type I collagen microfibers hydrated at pH 2.5, were compared with those of another low-density ink formulation, 175-200 μm type I collagen microfibers hydrated at pH 2.5, that successfully produced stable filaments (Supplementary Figure S3A&B). Although both formulations exhibited predominantly elastic behavior during a frequency sweep (G’ > G” across the tested frequency range), the <50 μm microfiber formulation demonstrated considerably lower G’ values of approximately 160-180 Pa at low strains during a strain sweep compared to the 175-200 μm microfiber formulation, which exhibited values of approximately 800-900 Pa at low strains. Consistent with the strain sweep, the 175-200 μm microfiber formulation also exhibited substantially higher G’ values across the tested frequency range.

The rheological properties of type I collagen and type II collagen microfiber-based inks were compared using formulations prepared at their respective isoelectric points with the same microfiber size-range, 175-200 μm, and 20% collagen density (Supplementary Figure S3C&D). At low strains during a strain sweep, even though the type II collagen formulation exhibited a higher G’, the type I collagen formulation exhibited a higher yield strain. In contrast, the type I collagen formulation during a frequency sweep exhibited a higher G’ across the tested frequency range, suggesting greater elastic strength over a broader range of deformation timescales.

In general, filaments extruded from the low-density collagen microfiber ink formulations prepared at pH 2.5 exhibited a greater tendency to fuse during printing than those produced from higher-density formulations. When comparing two formulations consisting of the same type I collagen microfiber size-range, 175-200 μm, but differing in collagen densities, 2.6% or 20.2%, the low-density formulation exhibited consistently lower G’ values during both frequency and strain sweeps, as well as lower yield strain and G’/G” crossover strain values during the strain sweep (Supplementary Figure S3E&F).

3.3 Construct Integrity: 4-Layers

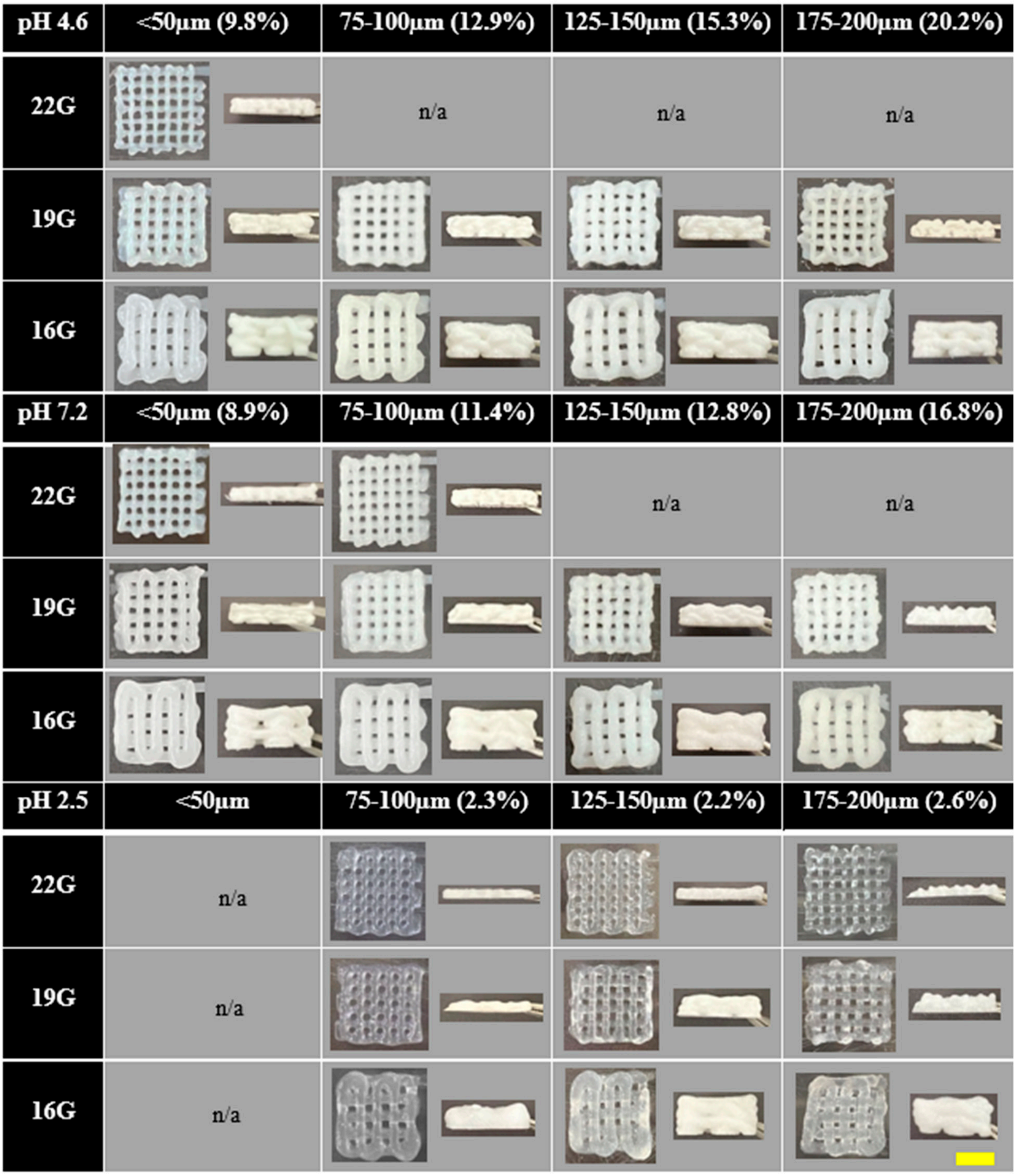

Figure S4: Printed 4-layer crosshatch patterns. Top-view images immediately after printing and side-view images after lyophilization of 4-layer crosshatch patterns printed with type I collagen microfiber-based inks of varying microfiber size-ranges hydrated at different pH and centrifuged at 14,000×g for 6 minutes. The average percent collagen content is included in parenthesis. Scale bar: 5mm.

3.4 Peripheral Pore Closure

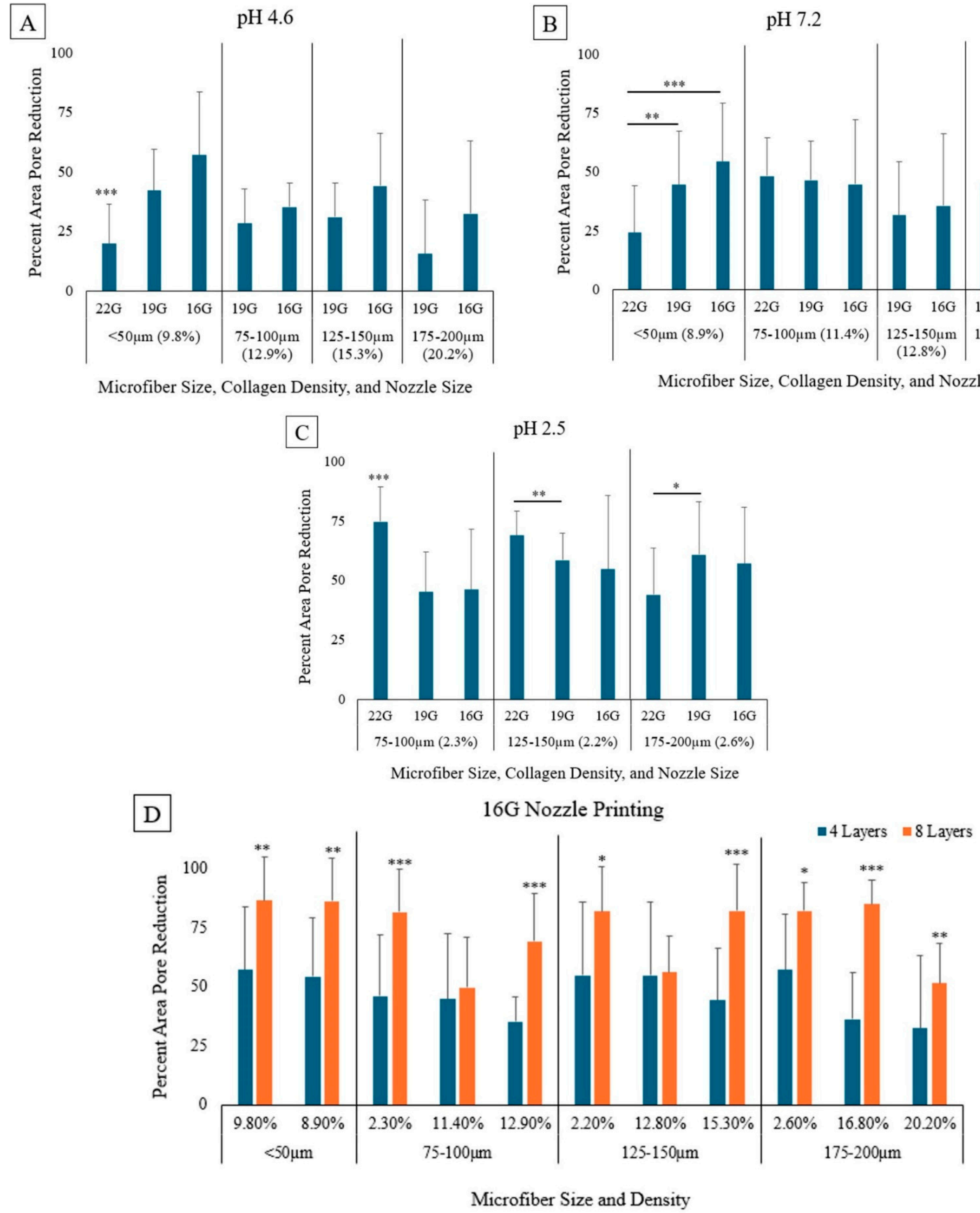

Figure S5: Pore closure analysis. Percent area pore reduction of pores on the periphery of crosshatch patterns printed for (A-C) 4 layers and for (D) 8 layers using a 16G nozzle.

4. SEM: Low Magnification Images

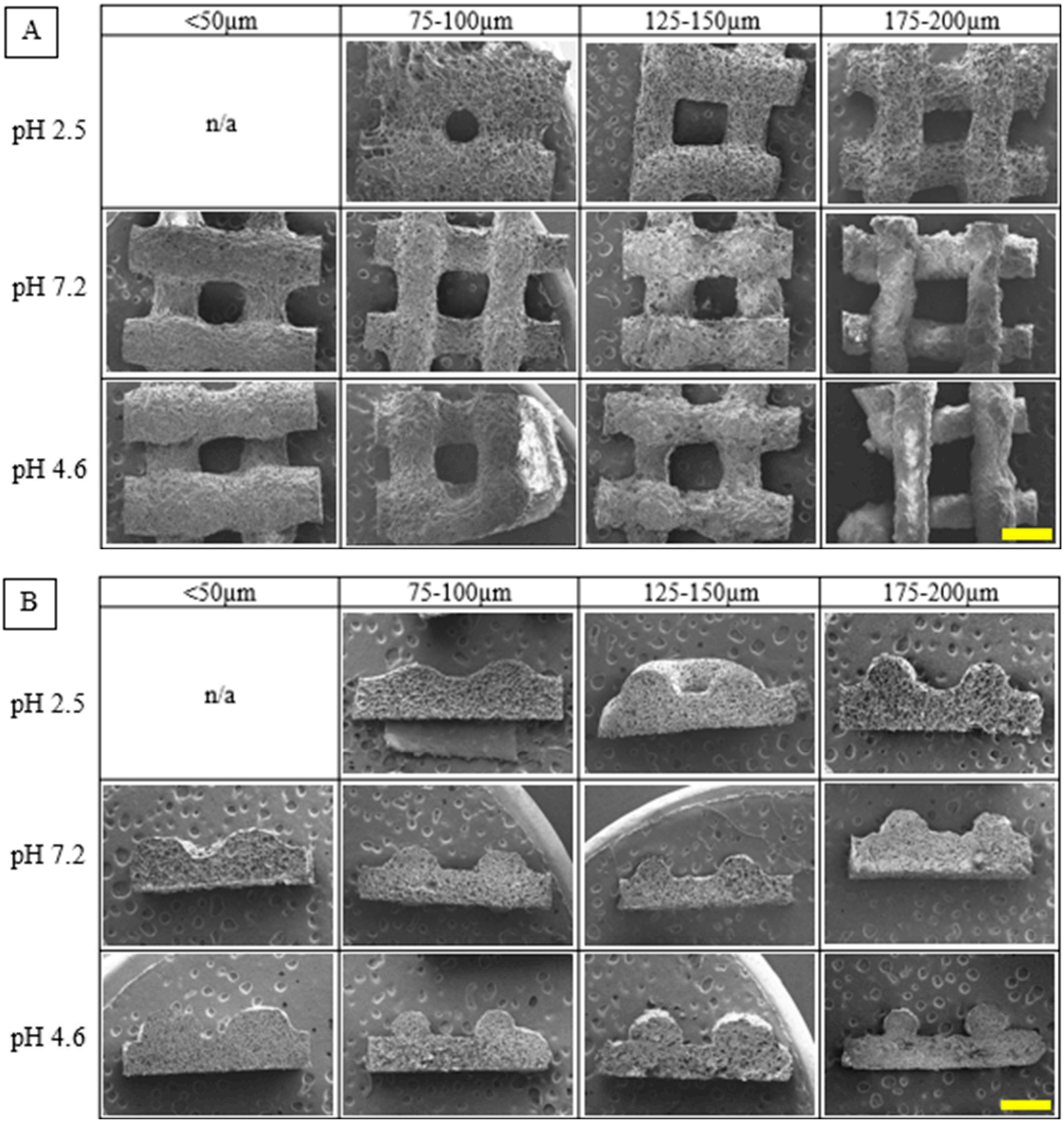

Figure S6: Low-magnification SEM images. 30× magnification of the (A) surface and (B) cross-section of lyophilized, 2-layer crosshatch patterns of type I collagen microfiber-based inks 3D-printed using a 19G nozzle from Figure 7. Scale bars: 1mm.

5. Type I Collagen + Bone Mineral Composite Ink for Bone Regeneration

The potential applications of printable bone mineral composite ink formulations were shown in Figure 11. A 60:40 bone mineral composite ink (with 10% microfibers 75-100µm in size hydrated in PBS) printed into a conical plug (10Ø × 15mm height) can be used for bone grafting purposes in the dental field. This ink was printed with a 19G blunt-tipped nozzle with a printing speed of 1000 mm/min and a calculated extrusion flow rate of approximately 10.9 µl/sec. An 80:20 bone mineral composite ink (with 10% microfibers 50-75µm in size hydrated in PBS) printed into a 5×2×0.5cm (L×W×H) solid, rectangular block can be used as a scaffold for spine fusion applications. This ink was printed with a 14G blunt-tipped nozzle with a printing speed of 250mm/min and a calculated extrusion flow rate of approximately 9.4µl/sec.

6. Type I Collagen Tube Fabrication

In a pilot study (Figure 12), tubes were fabricated from a 10% type I collagen microfiber-based ink which was prepared usings 25-50µm microfibers hydrated in 0.1M sodium acetate buffer (pH 4.6). The ink was extruded through a 20G blunt-tipped nozzle along a spinning rod (~290 RPM) at a printing speed of 50mm/min and a calculated extrusion flow rate of approximately 0.284µl/sec.

References

1. Zhou S, Capariño K, LeMarble P, Li ST. Developing insoluble type II collagen microfiber scaffolds for cartilage tissue engineering. Biomater Adv. 2026 Sep;186:214934. doi: 10.1016/j.bioadv.2026.214934. Epub 2026 May 7. PMID: 42127505.
2. Milazzo, M., Rovelli, R., Ricci, C., Macchi, T., Gallone, G., & Danti, S. (2025). Rheology and printability of hydroxyapatite/sodium alginate bioinks added with bovine or fish collagen peptides. *Gels*, *11*(3), 209.
